# Impact and Process Evaluation of Integrated Community and Clinic-Based HIV-1 Control: A Cluster-Randomised Trial in Eastern Zimbabwe

**DOI:** 10.1371/journal.pmed.0040102

**Published:** 2007-03-27

**Authors:** Simon Gregson, Saina Adamson, Spiwe Papaya, Jephias Mundondo, Constance A Nyamukapa, Peter R Mason, Geoffrey P Garnett, Stephen K Chandiwana, Geoff Foster, Roy M Anderson

**Affiliations:** 1 Department of Infectious Disease Epidemiology, Faculty of Medicine, Imperial College London, London, United Kingdom; 2 Biomedical Research and Training Institute, Harare, Zimbabwe; 3 Family AIDS Caring Trust, Mutare, Zimbabwe; 4 Faculty of Health Sciences Research and Postgraduate Studies, University of Witwatersrand, Johannesburg, South Africa; University of Amsterdam, Netherlands

## Abstract

**Background:**

HIV-1 control in sub-Saharan Africa requires cost-effective and sustainable programmes that promote behaviour change and reduce cofactor sexually transmitted infections (STIs) at the population and individual levels.

**Methods and Findings:**

We measured the feasibility of community-based peer education, free condom distribution, income-generating projects, and clinic-based STI treatment and counselling services and evaluated their impact on the incidence of HIV-1 measured over a 3-y period in a cluster-randomised controlled trial in eastern Zimbabwe. Analysis of primary outcomes was on an intention-to-treat basis. The income-generating projects proved impossible to implement in the prevailing economic climate. Despite greater programme activity and knowledge in the intervention communities, the incidence rate ratio of HIV-1 was 1.27 (95% confidence interval [CI] 0.92–1.75) compared to the control communities. No evidence was found for reduced incidence of self-reported STI symptoms or high-risk sexual behaviour in the intervention communities. Males who attended programme meetings had lower HIV-1 incidence (incidence rate ratio 0.48, 95% CI 0.24–0.98), and fewer men who attended programme meetings reported unprotected sex with casual partners (odds ratio 0.45, 95% CI 0.28–0.75). More male STI patients in the intervention communities reported cessation of symptoms (odds ratio 2.49, 95% CI 1.21–5.12).

**Conclusions:**

Integrated peer education, condom distribution, and syndromic STI management did not reduce population-level HIV-1 incidence in a declining epidemic, despite reducing HIV-1 incidence in the immediate male target group. Our results highlight the need to assess the community-level impact of interventions that are effective amongst targeted population sub-groups.

## Abstract 2

In cluster-randomised trial in Zimbabwe integrated peer education, condom distribution, and management of sexually transmitted infections did not reduce incidence of population-level HIV-1.

## Editors' Summary

**Background.:**

Sub-Saharan Africa has been hit heavily by HIV/AIDS, and Zimbabwe in particular has been very badly affected, with over one-fifth of its adult population infected with HIV. However, this proportion has been declining slowly in recent years, and the same trend has also been seen in a few other African countries. It is not clear whether these trends are related to changes in the way people behave, perhaps as a result of public health and prevention campaigns, or rather are due to changes in the natural spread of the HIV epidemic. However, there is considerable uncertainty about how we should carry out campaigns that try to get people to change their behavior. One possible approach for achieving behavior change involves peer education: that is, education carried out within the community, by at-risk community members themselves. Another approach involves tying together a set of related programs that deliver information and education through health clinics and directly in the community. Such programs are termed “integrated community and clinic-based HIV prevention.”

**Why Was This Study Done?:**

The researchers wanted to find out whether providing integrated community and clinic-based strategies for HIV prevention in Eastern Zimbabwe could reduce the proportion of people within the community infected with HIV. If successful, then the strategies could be effective elsewhere, for example in other African countries where behavior patterns and the HIV epidemic are similar to the situation studied here.

**What Did the Researchers Do and Find?:**

The research was done as a cluster-randomized trial. This means that different communities were assigned by chance to one of two trial arms, either an “intervention arm”, where the community and clinic-based strategies would be delivered, or a “control” arm which would not have additional services. Six pairs of communities in Eastern Zimbabwe were compared, each of which had its own health center. Control communities received the standard government services for preventing HIV. The other communities received a package of various additional strategies. These included education and condom distribution amongst sex workers and their clients; better services at sexually transmitted infection (STI) clinics (STIs can increase the risk of HIV infection); and educational HIV/AIDS open days at health centers. The researchers planned to compare, between the two arms, the number of people who became infected with HIV over the course of the trial. They found that there was no statistical difference in the number of people in the intervention arm who became infected with HIV over the course of the trial, as compared to people in the control arm. Men in the intervention communities were more likely to have effective treatment for STIs, but women were more likely to show risky behaviors, such as having sex at a younger age, and having unprotected sex. However, men in the intervention communities were more knowledgeable about HIV/AIDS than men in the control communities. One strategy in the intervention arm (delivery of education and condom distribution among sex workers and their clients) may have been less successful because of the economic situation at the time, which meant that the income-generating projects that were supposed to support this initiative were impossible.

**What Do These Findings Mean?:**

Some of the results from this trial are encouraging, for example an improvement in male participants' knowledge and behavior. However, overall, the intervention did not have an impact on the HIV infection rate in the community. Some other trials have also shown similar results. These results mean that other strategies need to be developed, and tested, which will encourage people to change their behavior patterns and reduce the risk of getting HIV. However, trials such as this are very difficult to design, carry out, and interpret. In particular, if a complex intervention such as this fails, it is often hard to tell whether it did so because the intervention was not delivered successfully, or because it did not work.

**Additional Information.:**

Please access these Web sites via the online version of this summary at http://dx.doi.org/10.1371/journal.pmed.0040102.

- Information from Avert, an international HIV/AIDS charity, on HIV and AIDS in Zimbabwe
- Information from UNAIDS, the United Nations Programme on HIV/AIDS, on strategies for HIV prevention
- HIV/AIDS minisite from the World Health Organization
- The Web site of the Manicaland HIV/STD Prevention Project discusses this project

## Introduction

HIV-1–prevalence declines may now be occurring in some sub-Saharan African countries [1]. However, there remains little direct evidence that prevention measures—rather than natural HIV-1 epidemic dynamics [2] or behaviour change prompted by mortality [3]—have contributed to the slowing of HIV-1 epidemics [4,5]. Syndromic management of sexually transmitted infections (STIs) proved effective early in an HIV-1 epidemic in north-west Tanzania [6]. Peer education to promote safe behaviours showed promise in early process evaluations [7], but a randomised controlled trial (RCT) of factory workers in Harare, Zimbabwe, done in the mid-1990s, proved inconclusive [8]. Subsequent RCTs of syndromic management [9] and mass treatment of STIs [10], together with an information, education, and communication (IEC) behaviour-change programme [9], showed no effect in more mature epidemics.

Integrated implementation of synergistic community-based HIV-1 control strategies could be a more cost-effective and sustainable approach to HIV-1 prevention than parallel application of vertical (top-down) programmes [11]. One scientific evaluation of such a strategy has been reported in which a combination of IEC activities amongst the general population and syndromic STI management showed no impact on HIV-1 incidence at the population level [9], although participation in the IEC activities was associated with reduced HIV-1 infection in women [12].

We conducted a cluster-RCT to test the hypothesis that integrated implementation of combined community- and clinic-based HIV-1 prevention, in which IEC activities focus primarily on high-risk populations, can be feasible and effective in reducing HIV-1 incidence in a major maturing epidemic in eastern Zimbabwe (Protocols S1 and S2; Text S1 and S2).

## Methods

### Participants and Randomisation Procedure

**Figure 1:**
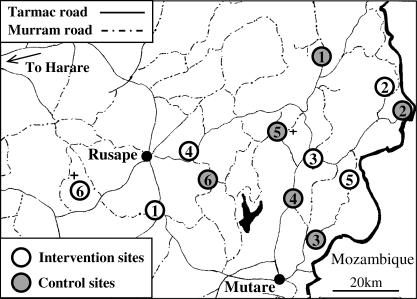
Location of Intervention and Control Communities in Manicaland Province, Eastern Zimbabwe

The study communities comprised six pairs of communities matched by socio-economic type—small town, tea/coffee estate, forestry plantation, roadside trading settlement, and subsistence farming area (two pairs) (Figure 1). Each community included at least one Government or Mission health centre. It was anticipated that HIV-1 incidence would be similar within each pair of communities. Within each pair, one community was assigned at random (un-blinded coin toss by a Ministry of Health official witnessed by programme and research personnel) to receive the additional intervention and the other to be the control. These procedures were designed to ensure that Mission, non-governmental organisation, and private sector programmes (for details, please refer to the following section) would be distributed evenly between intervention and control sites.

We assessed the effect of the intervention using results from laboratory tests for HIV-1 infection and questionnaire data collected in the baseline and 3-y follow-up rounds of a population-based, closed-cohort survey. The 12 study communities were enumerated in a phased manner, with paired communities being enumerated consecutively to minimise the effects of any seasonal factors. HIV-1–prevention activities were commenced in each intervention community shortly after completion of the baseline survey in that community. In each community, individuals eligible for the study were identified in the first round using data from household listings prepared in an initial census. All males and females aged 17–54 y and 15–44 y at last birthday (the age groups expected to have the highest incidence of HIV infection), respectively, who had slept in a household in the community for at least four nights in the previous month, and who had also done so at the same time 1 y earlier, were considered eligible for the study. In heterosexually driven HIV-1 epidemics, risk of infection can be correlated amongst marital partners [13]. Therefore, to maximise statistical power to detect differences in HIV-1 incidence, enrolment was restricted to one randomly selected member per marital group.

### Interventions

Intervention and control communities were to receive standard Government services including basic syndromic STI management, condom distribution from health clinics and Zimbabwe National Family Planning Council outlets, home-based care, and limited HIV/AIDS–focussed IEC activities (e.g., occasional AIDS-awareness meetings and distribution of posters and leaflets). In addition, social marketing of male and female condoms would be provided through an ongoing national programme [14].

The intervention comprised targeted and population-level strategies to promote safer sexual behaviour and to improve treatment of STIs that facilitate HIV-1 transmission. The intervention strategies were implemented by two local non-governmental organisations (Family AIDS Caring Trust and the Biomedical Research and Training Institute) and the Zimbabwe Ministry of Health and Child Welfare through an integrated programme of community- and clinic-based activities. Integration of the individual programme components was achieved through the joint involvement of the participating agencies in the planning and implementation of activities and through the inclusion of biomedical and behavioural aspects within each component. The programme design comprised three key components: (1) peer education and condom distribution amongst commercial sex workers and male clients at workplaces and in the general community, supported by income-generating projects; (2) strengthened syndromic management of STI services at local health centres; and (3) open days with HIV/AIDS IEC activities at health centres to promote safer sexual behaviour and to increase the uptake of local STI treatment services.

The peer-education component was based on a model which had been developed by the Project Support Group at the University of Zimbabwe [7] and which had been widely implemented within Zimbabwe and neighbouring countries. Activities were held weekly at workplaces and at locations within the general community (e.g., beer halls and markets) where casual relationships were most frequently formed [15]. The target population comprised sex workers and male clients who form a bridge population in HIV transmission [16] between sex workers and the monogamous (or serial monogamous) majority of women [17,18]. It was posited that the high HIV-1 incidence observed amongst young women could be reduced by altering the behaviour of their older male partners whose own behaviour was intrinsically more risky [19]. The behavioural component would be reinforced in counselling sessions with STI patients and through micro-credit income-generating projects to reduce unmarried women's dependence on commercial sex work. The micro-credit scheme consisted of small interest-free loans repayable over 10 mo, provided to groups and to individuals together with training in small-business management. The targeted activities would be extended to the general population through open days held at local health centres.

Besides providing basic HIV/AIDS information, it was envisaged that programme meetings and activities, by their continuous nature, would sustain high levels of awareness of the risks of HIV transmission and would facilitate renegotiation of community social norms, making safer behaviours easier to adopt. The key messages of the programme were: (1) remain faithful to one regular sexual partner; (2) use condoms consistently with any casual sexual partners; and (3) seek prompt and effective treatment for any STIs.

Syndromic management of STIs at primary healthcare centres was first introduced in Zimbabwe in the 1980s [20] and formed the basis of STI diagnosis and treatment services at baseline in the intervention and control communities. It was envisaged that these services could be strengthened and made more effective through a programme of regular classroom training and on-site supervision of nursing staff, through the introduction of training in systemic counselling for STI patients, and through the provision of small quantities of treatment drugs to cover delays in routine supplies.

Quality-assurance procedures applied in the intervention communities included pre- and post-training tests for peer educators and, for nursing staff, attending the syndromic STI management and systemic counselling courses, regular on-site supervision (including random spot checks) and training, refresher courses, routine planning meetings and monitoring of service statistics, and quarterly workshops where detailed programme procedures were reviewed and updated. An interim qualitative process evaluation of intervention activities was conducted during the inter-survey period, and a report on the findings was provided to the implementing organisations.

### Outcome and Process Measures

The primary outcome of the study was HIV-1 incidence at the community level amongst individuals who were uninfected at baseline. Blood was collected onto Whatman No. 3 filter paper and transported to the Biomedical Research and Training Institute laboratory in Harare. Blood spots were air dried at 4 °C and, for long-term (>1 mo) storage, were kept at −20 °C. For baseline studies, blood was eluted into phosphate-buffered saline, and antibodies to HIV were detected using a dipstick dot EIA (ICL-HIV-1/HIV-2 Dipstick, [PATH, http://www.path.org; produced locally in Thailand]) and a standard protocol [21,22]. All positive results and a 10% sample of negative results were confirmed using a plate EIA (Abbott Third-Generation HIV-1/HIV-2 EIA [http://www.abbott.com] or Genelavia MIXT HIV-1/HIV-2 [Sanofi Diagnostics Pasteur, Marnes La Coquette, France]). At follow-up, a similar protocol was followed. Only the samples from those participants recorded as being HIV seronegative at baseline were tested at follow-up, again using a dot EIA (ICL-HIV-1/HIV-2 Dipstick, [PATH, produced locally in India]). Where seroconversion was indicated, the frozen stored baseline sample was retested to confirm the original negative result using the same dot EIA test. Where the baseline result remained negative, the Abbott EIA test was used to confirm both baseline and follow-up results. The change in place of manufacture of the dot EIA and the exclusive use of Abbott test kits to confirm positive sera at follow-up was due only to changes in the supply of test reagents, and not to perceived changes in sensitivity or specificity [23]. Apart from the principal investigators (based in Harare, London and Oxford) and those nurses given permission by participants requesting voluntary counselling and testing (VCT), all research personnel remained blind to the HIV-1 status of individual participants.

Secondary outcomes, measured at the community and individual level, were self-reported genital ulcers and urethral or vaginal discharge in the past year (STI cases), STI treatment effectiveness (self-reported cessation of symptoms), indicators of sexual and health-seeking behaviour change, and HIV/AIDS knowledge. The behaviour-change variables assessed were sexual debut, sexual partner change in the past year, non-regular partnerships in the past month, and unprotected sex with regular and casual partners in the past 3 y. The data on sexual partnerships and condom use were collected using the Informal Confidential Voting Interview method for 75% of respondents selected at random in the first round of the survey. This method includes procedures to build rapport, ensure a non-judgemental interview approach, and provide reassurance that there are no right or wrong answers to questions of a personal nature, and uses a simple secret voting-box system to reduce embarrassment and guarantee confidentiality in low-development settings [18]. Its use has been shown to be associated with greater disclosure of socially proscribed behaviour in the study population [24].

Process indicators examined comprised changes in knowledge and psychosocial status and indicators of programme coverage and quality.

### Sample-Size Calculations

Initial sample-size calculations assumed 20% HIV-1 prevalence at baseline, 30% loss to follow-up after 2 y, and 80% power to detect a 40% reduction in HIV-1 incidence in the intervention communities compared with control communities, assuming a background yearly incidence of 2%. Based on six pairs of communities and a co-efficient of variation between communities of 0.15, the required sample size in each community was 1,000. Funding constraints and slower than anticipated implementation of intervention activities led to revisions of the sample size for each community to 800 and the length of follow-up to 3 y, respectively. Assuming a proportionate increase in loss to follow-up to 41%, these arrangements also yielded 80% power to detect a 40% reduction in HIV-1 incidence.

### Statistical Methods

To test the randomisation with small numbers of communities, HIV-1 prevalence, STI history, and socio-demographic characteristics were compared at baseline for study participants in the intervention and control communities, together with uptake of STI treatment and VCT services offered at baseline.

Outcome and process indicators were compared for intervention versus control communities. Analysis of the primary outcome was on an intention-to-treat basis. Incident events and person-years at risk of seroconversion were used to calculate HIV-1 incidence rates and unadjusted and adjusted incidence rate ratios (IRR) with 95% confidence intervals (CIs) for each pair of communities. Adjustment was made for sex, 3-y age group, and community-level baseline HIV prevalence. The overall IRRs (unadjusted and adjusted) were taken to be the geometric means of the IRRs for the six pairs of communities. We calculated 95% CIs for each geometric mean as geometric mean ± 1.96 × standard error of the geometric mean. Paired student *t-*tests on the logarithms of the pair-specific IRRs were used to test whether these differed significantly from unity [25]. The coefficient of variation between communities was calculated based on baseline HIV prevalence using a standard procedure for pair-matched studies [26].

Analyses of prevalence for secondary outcome and process variables were conducted separately for male and female respondents seen at both survey rounds by fitting logistic regression models to the individual-level data and adjusting for community pair and, where available, value of variable at baseline.

Since most programme activities were targeted and overall coverage of programme activities was therefore limited, sub-group analyses, adjusted for community pair, were done for HIV-1 incidence and behavioural outcomes to assess the individual-level effects of attendance at programme meetings.

Data were entered and validated using SPSS-PC (http://calcnet.mth.cmich.edu/org/spss/index.htm) and data analysis was conducted in Stata version 7 (http://www.stata.com). Statistical tests were double-sided and results were taken to be significant at the 5% level.

### Ethical Approval

All study participants in the intervention and control communities were offered free VCT for HIV-1, an information sheet on HIV/AIDS, results from a diagnostic test for Trichomonas vaginalis [27] (done at baseline only), and free treatment for T. vaginalis and other STIs from a research nurse. Testing and treatment for T. vaginalis was provided because the prevalence of other curable STIs was low in the study areas [22]. Antibodies reactive with T. vaginalis were detected in DBS eluates following a previously described procedure [27,28].

Written informed consent was sought as a condition of enrolment and continuation in the study. Prior ethical approval was obtained from the Research Council of Zimbabwe, number 02187; the Applied and Qualitative Research Ethics Committee in Oxford, United Kingdom, N97.039; and the UNAIDS Research Ethics Committee, ERC 98/03.

## Results

### Participant Flow

**Figure 2:**
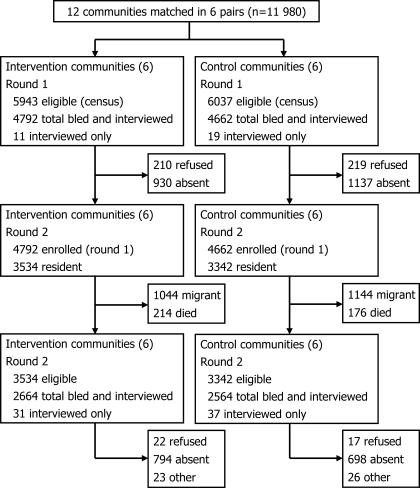
Flow-Chart Comparing Participation and Follow-Up Rates in the Intervention and Control Communities Individuals enrolled in round 1 and still resident in the study communities were considered eligible for participation in round 2.

In round 1 of the census (July 1998 to February 2000), 5,943 and 6,037 eligible individuals in the intervention (total population size 18,104) and control (18,633) communities, respectively, were selected for recruitment into the study cohort (Figure 2). In round 2, 3 y later (July 2001 to February 2003), 1,044 (23%) and 1,144 (26%) of baseline respondents who were still alive had migrated away from the intervention and control communities, respectively, and were therefore lost to follow-up (Figure 2). At both baseline and follow-up, migrants and non-migrants had similar risks of HIV-1 infection and associated behaviour [29]. Of those still resident in the intervention and control communities, 2,664 (75%) and 2,564 (77%), respectively, were interviewed and blood samples taken for a second time. Temporary absence from the usual place of residence was the main reason for non-participation in the intervention (*n* = 794, 95%) and control (*n* = 698, 94%) communities. The overall proportions of baseline respondents followed up at the end of the study were 55% and 56% in the intervention and control communities, respectively. The median follow-up of communities was 3.0 y (range of median within communities, 3.0–3.1).

### Baseline Data

HIV-1 prevalence was higher in the intervention communities than in the control communities (24% versus 21%, risk ratio 1.13 [95% CI 1.05–1.22], *p* = 0.001). T. vaginalis infection, secondary school education, and spatial mobility were more common in the control communities, whilst history of genital discharge and uptake of STI treatment and VCT services offered in the survey were low overall but more frequent in the intervention communities (Table 1). However, the differences in each case were small and were unlikely to be clinically meaningful.

**Table 1:**
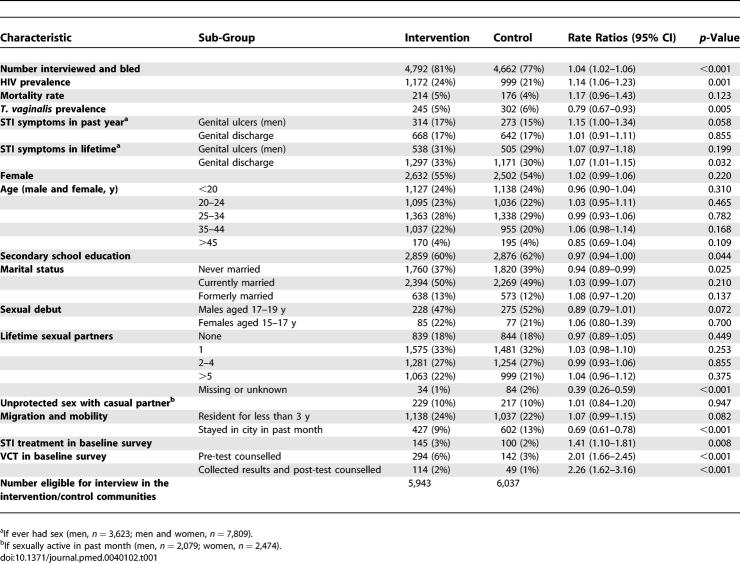
Baseline Characteristics of the Study Populations

### Outcomes and Estimation

Median follow-up per person was 2.9 y (range 1.4–3.9) and 3.0 y (range 1.5–4.1) in the intervention and control communities, respectively. In total, 4,052 individuals had 212 incident events of HIV-1 during 12,009 person-years at risk, giving an HIV-1 incidence rate of 1.77 per 100 person-years at risk. HIV-1 incidence was higher in communities with higher baseline HIV prevalence (IRR 11.49 [95% CI 1.80–73.40], *p* = 0.010), but this difference disappeared after adjustment for stratification by community type (*p* = 0.8). HIV-1 incidence was higher in the intervention communities than in the control communities overall, and in each community type, except in the forestry plantations where it was almost identical (Table 2). The difference was not significant after adjustment for sex, age group, and baseline HIV prevalence (IRR 1.27 [95% CI 0.92–1.75], *p* = 0.012). The observed coefficient of between-community variation was 0.14.

**Table 2:**
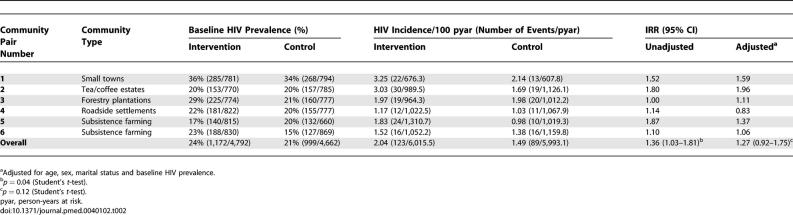
HIV Prevalence at Baseline and HIV Incidence and IRRs for Intervention Versus Control Communities

Looking at outcome indicators for community members (rather than for communities—the unit of randomisation), self-reported STI symptoms were similar in both sets of communities (Table 3). Treatment for STI symptoms in males was effective more frequently in the intervention communities, with men in the intervention community in five of the six matched pairs reporting reduced symptom recurrence. However, more young women in the intervention than in the control communities had started sex, and reports of unprotected sex with a casual partner in the study period were more common in the intervention communities. No differences were observed in consistent condom use with regular partners between the two sets of communities. In the intervention communities, knowledge about HIV/AIDS was enhanced amongst men, and more respondents reported a close relative or family member with AIDS (sex- and age-adjusted prevalence odds ratio 1.22 [95% CI 1.05–1.42], *p* = 0.009). Slightly more women in the intervention communities reported that condom use within marriage was becoming acceptable, but a greater proportion of men agreed with the statement that “condoms reduce the pleasure of sex”.

**Table 3:**
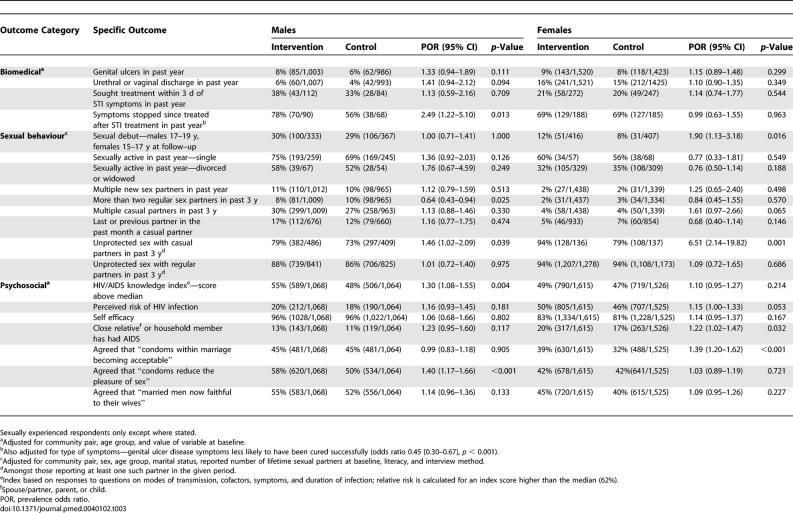
Biomedical, Sexual Behaviour, and Psychological Outcomes at Follow-up by Residence in the Intervention and Control Communities

A total of 63,261 peer-education meetings were held, and 6.8 million condoms were distributed by the programme in the intervention communities (Table 4). Outputs increased over time as new communities entered the programme. However, owing to high inflation and economic decline, the micro-credit income-generating projects proved impossible to implement. We were able to obtain data on STI episodes treated at clinics in the 11 out of 12 study communities that reported cases to the administrative districts of Mutasa and Makoni. In the three intervention communities each in Mutasa and Makoni, STI cases fell by 66% and 51%, respectively, over the 3-y study period. Similar declines of 67% and 52% occurred at clinics in the four control communities in Mutasa and the one control community in Makoni. Coverage of training in syndromic STI management and systemic counselling for nursing staff was high (Table 4).

**Table 4:**
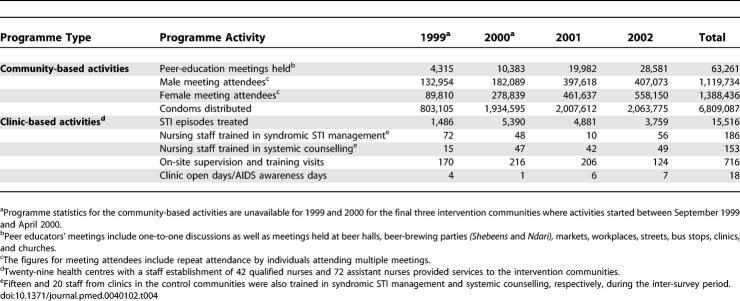
Summary of Service Statistics on Programme Output

Most of the activities were targeted at high-risk groups. In the general population sample interviewed in the follow-up survey, 1,779 (35%) and 647 (13%) of 5,098 respondents reported attending an HIV/AIDS meeting and a programme meeting, respectively (Table 5). More respondents in the intervention communities than in the control communities attended an HIV/AIDS meeting (41% versus 28%, prevalence rate ratio 1.44 [95% CI 1.33–1.56], *p* < 0.001) and a programme meeting (20% versus 5%, 4.27 [95% CI 3.52–5.17], *p* < 0.001), and participation was higher among men than women (prevalence rate ratio 1.32 [95% CI 1.14–1.53], *p* = 0.002). Fewer women in the intervention communities had heard about HIV/AIDS from external sources or believed that STI drugs were available at their local clinics. Sixty-two (2%) out of 2,528 respondents in the control communities reported spending at least 1 d in the past month in the intervention communities; the equivalent number for respondents in the intervention communities visiting control communities was 70 (3%) out of 2,683.

**Table 5:**
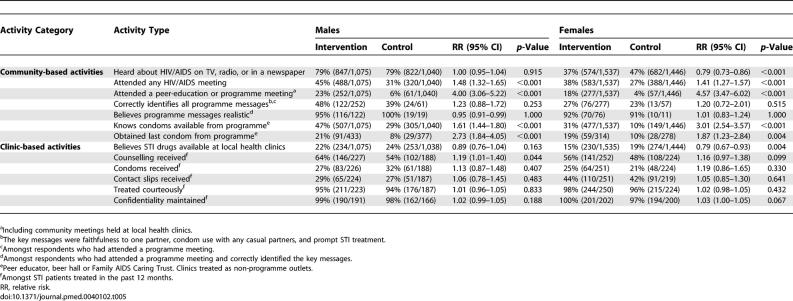
Intervention Coverage

### Ancillary Analyses

In exploratory analysis to assess where the intervention failed, we found that HIV-1 incidence was reduced in males (IRR 0.48 [95% CI 0.24–0.98], *p* = 0.044) who reported attending programme meetings, after adjustment for the targeting of activities to groups with high-risk behaviour (Table 6). Amongst men who reported one or more casual sexual partners in the past 3 y, fewer of those who attended meetings reported unprotected sex with these partners (prevalence odds ratio 0.45 [95% CI 0.27–0.75], *p* = 0.002). HIV-1 incidence was not associated with programme participation in women.

**Table 6:**
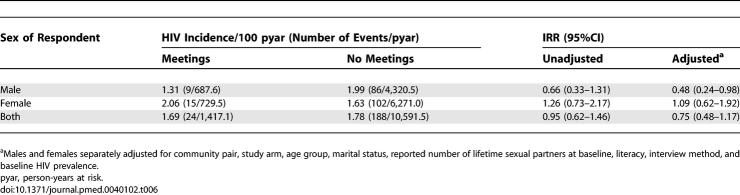
HIV Incidence and IRRs by Meeting Attendance and Sex

## Discussion

### Interpretation

We conducted a scientific trial of the feasibility and impact of an integrated community- and clinic-based HIV-1–prevention intervention. The income-generating projects apart, the intervention activities were feasible. The outputs of the programme were extensive with more than 63,000 meetings being conducted and almost 7 million condoms distributed by trained peer educators. Programme messages were considered relevant and realistic. Local STI treatment and counselling services were strengthened and promoted in accordance with the intervention protocol. For male participants, these activities improved HIV/AIDS knowledge, increased the effectiveness of STI treatment, increased consistent condom use with casual partners, and reduced HIV-1 incidence. However, the cluster-RCT results clearly show that the intervention had no positive impact at the community level and suggest possible detrimental effects on the onset of female sexual activity and condom use with casual partners over a 3-y timeframe.

Did the cluster-RCT design fail to capture the true effect of the intervention? There are three possibilities: (1) inadequate statistical power; (2) insufficient follow-up; and (3) contamination of intervention within control communities. The study design provided adequate statistical power to detect a meaningful average reduction (40%) in HIV-1 incidence in the intervention versus the control communities over a 3-y observation period. In hindsight, an effect size of 40% was too optimistic and the study had insufficient power to detect a smaller effect. However, there was no trend in the results towards reduced HIV-1 incidence in the intervention communities. Largely due to migration, attrition was close to that anticipated in the study design and was comparable to other recent cohort studies [6,10,9,30]. Migrants had similar characteristics and sexual behaviour to non-migrants [29].

The results of the exploratory sub-group analysis generate the hypothesis that high-risk behaviour was reduced in males attending programme meetings but did not translate into a wider impact on HIV-1 incidence at the population level. Changes in core and bridge populations may take more time to reflect in the general population than was observed in the trial. However, a longer period of follow-up would have increased attrition, and the finding of a possible adverse effect at the population level meant that it would not have been ethical to continue with the same intervention. Future trials of behaviour-change interventions may need to include multiple rounds with phased recruitment and (where interim results are favourable) may need to consider phased intervention implementation.

We minimised intervention contamination by selecting physically separated study communities, and movements between intervention and control communities were rare. However, a similar peer-education programme was implemented in one control community (small town), and HIV-1–prevention activity was considerable in all control communities that also had greater access to information from external sources. In some cases, programme messages (e.g., promotion of condom use) conflicted with those of other agencies working in the intervention communities. The effects of these other programmes could have limited our ability to detect a reduction in HIV-1 incidence caused by the current intervention.

The absence of an observed effect of the intervention was not explained by differences in HIV-1 prevalence, sexual behaviour, STI cofactors, mobility, or socio-demographic composition at baseline. The earlier sexual debut in females and greater unprotected sex with casual partners seen in the intervention communities during the study period were not present at baseline but could reflect increased willingness to report high-risk behaviours in settings where there was more open discourse about HIV and AIDS.

The peer-education programme could have had some effect for male but not for unmarried female participants. Preliminary findings from subsequent qualitative investigations indicate that, in the predominantly rural communities in which the study was conducted, poverty and the associated failure of income-generating projects meant that some peer educators were unable to maintain safer behaviours. Given their increased visibility within the community—intended to enhance their status and self-esteem and, thus, to reinforce their commitment to and role as models for behaviour change—they may, inadvertently, have served as negative role models and, thereby, may have contributed to the greater female early-age sexual activity. Free distribution of condoms by women still engaging in unprotected commercial sex led to their being poorly valued and reinforced their association with promiscuity.

### Generalisability of Findings

Epidemiological context can affect the impact of interventions [31], and structural obstacles can limit the pace and extent to which activities are implemented and the quality of these activities [32]. The HIV-1 epidemic stabilised in eastern Zimbabwe during the study period, with HIV-1 prevalence declining by 40%–50% in young adults [23]. This decline was accompanied by delayed sexual debut, reduced sexual partner change, and consistent condom use with casual partners [33,23]. Prevalence of syphilis, gonorrhoea, and Chlamydia is low, but non-curable herpes simplex virus type 2 remains common [22]. Risk reduction makes transmission more fragile, and an intervention could have a larger effect when set against secular behavioural changes [2]. Mathematical model simulations suggest that there would also be a greater chance of detecting a significant effect of the intervention even though there would be fewer seroconversions to power the calculation [34,35]. Structural obstacles to intervention implementation included HIV/AIDS mortality which disrupted the programme by claiming the lives of two programme coordinators and several of the nursing staff and peer-educators. Economic decline made the income-generating projects unfeasible and reduced the effectiveness of other components of the intervention. We believe that the coverage of the peer-education programme was satisfactory, given the focus on highly sexually active individuals. Meeting coverage could have been under-estimated in the survey since one-to-one discussions and activities at beer halls and other public places may not have been recognised as meetings by those present. However, the high level of spatial mobility limited the number of people who were reached at the required level of intensity and consistency, whilst national shortages of foreign currency restricted fuel and drug supplies, hampered attempts to extend community activities into the more remote rural areas, and disrupted the STI treatment programme in both the intervention and control communities.

The intervention that we evaluated could have greater effect where an HIV-1 epidemic is younger, HIV-1 incidence is greater, local sexual networks are less diffuse, background STI control is weak, herpes simplex virus type 2 is less common, population mobility is lower, and/or the socio-economic climate is stable. We cannot rule out an effect of peer education in the urban intervention community since similar activities were implemented in the control community. Targeted peer education may work better in towns where bar-based sex work is more extensive. The absence of reduced HIV-1 incidence in farming estates reinforces doubts raised by the Harare factory workers study [8] concerning the efficacy of workplace peer education.

### Overall Evidence

These findings are important since the strategies evaluated—i.e., peer education, condom distribution, and syndromic STI management—are theory-based, have the potential for independent effects [11], and are widely promoted [36,37]. Syndromic STI management was effective in a nascent epidemic [6]. However, our disappointing findings echo those from recent trials [9,12] and emphasise the need for alternative strategies of behaviour-change promotion. Social marketing of condoms [14], larger poverty-alleviation programmes to reduce women's reliance on sex work—based on skills training and careful market research rather than on small-scale income-generating projects—and strategies which reach beyond high-activity core groups, such as the Popular Opinion Leader programme [38,39], and client-centred counselling [40], could be more viable and effective in reducing HIV-1 transmission in rural areas. Given the necessary economic conditions, unmarried women may still play a useful role in bar-based programmes since beer halls remain foci for high-risk behaviour [41,15].

## Supporting Information

### Trial Registration

This trial has the registration number ISRNCT00390949 in the International Standard Randomized Controlled Trial Number Register.

Found at: http://www.clinicaltrials.gov/ct/show/NCT00390949?order=1

Protocol S1 Protocol (35 KB DOC)

Protocol S2 Revisions to Protocol (35 KB DOC)

Text S1 CONSORT Checklist (48 KB DOC)

Text S2 Ethical Approval, Information Letter, and Consent Forms (2.8 MB PDF)

## Abbreviations

CI: confidence interval
IEC: information, education, and communication
IRR: incidence rate ratio
RCT: randomised controlled trial
STI: sexually transmitted infection
VCT: voluntary counselling and testing

## References

[pmed-0040102-b001] Asamoah-Odei E, Garcia Calleja JM, Boerma JT (2004). HIV prevalence and trends in sub-Saharan Africa: No decline and large subregional differences. Lancet.

[pmed-0040102-b002] Anderson RM, May RM (1991). Infectious diseases of humans: Dynamics and control.

[pmed-0040102-b003] Watkins SC (2005). Navigating the AIDS epidemic in rural Malawi. Popul Dev Rev.

[pmed-0040102-b004] UNAIDS (1999). Trends in HIV incidence and prevalence: Natural course of the epidemic or results of behaviour change?.

[pmed-0040102-b005] Stephenson JM, Obasi A (2004). HIV risk reduction in adolescents. Lancet.

[pmed-0040102-b006] Grosskurth H, Mosha F, Todd J, Klokke A, Senkoro K (1995). Impact of improved treatment of sexually transmitted diseases on HIV infection in rural Tanzania: Randomised controlled trial. Lancet.

[pmed-0040102-b007] Dube N, Wilson D, Williams B, Campbell C (1996). Peer education programs among HIV-vulnerable communities in Southern Africa. HIV/AIDS management in southern Africa: Priorities for the mining industry.

[pmed-0040102-b008] Machekano R, McFarland W, Mbizvo MT, Bassett MT, Katzenstein D (1998). Impact of HIV counselling and testing on HIV seroconversion and reported STD incidence among male factory workers in Harare, Zimbabwe. Cent Afr J Med.

[pmed-0040102-b009] Kamali A, Quigley M, Nakiyingi JS, Kinsman J, Kengeya-Kayondo J (2003). Syndromic management of STIs and behaviour change interventions on transmission of HIV-1 in rural Uganda: A community randomised trial. Lancet.

[pmed-0040102-b010] Wawer MJ, Sewankambo NK, Serwadda D, Quinn TC, Paxton LA (1999). Control of sexually transmitted diseases for AIDS prevention in Uganda: A randomised community trial. Lancet.

[pmed-0040102-b011] Garnett GP (2005). The role of herd immunity in determining the impact of sexually transmitted disease vaccines. J Infect Dis.

[pmed-0040102-b012] Quigley M, Kamali A, Kinsman J, Kamulegeya I, Nakiyingi JS (2004). The impact of attending a behavioural intervention on HIV incidence in Masaka, Uganda. AIDS.

[pmed-0040102-b013] Carpenter LM, Kamali A, Ruberantwari A, Malamba SS, Whitworth JAG (1999). Rates of HIV-1 transmission within marriage in rural Uganda in relation to the sero-status of the partners. AIDS.

[pmed-0040102-b014] Meekers D (2001). The role of social marketing in STD/HIV protection in 4600 sexual contacts in urban Zimbabwe. AIDS.

[pmed-0040102-b015] Lewis JJC, Garnett GP, Mhlanga S, Nyamukapa CA, Donnelly CA (2005). Beer halls as a focus for HIV prevention activities in rural Zimbabwe. Sex Transm Dis.

[pmed-0040102-b016] Morris M, Podhisita C, Wawer MJ, Handcock MS (1996). Bridge populations in the spread of HIV/AIDS in Thailand. AIDS.

[pmed-0040102-b017] Gregson S, Chandiwana SK (2001). The Manicaland HIV/STD Prevention Project: Studies on HIV transmission, impact and control in rural Zimbabwe. Zimbabwe Sci News.

[pmed-0040102-b018] Gregson S, Zhuwau T, Ndlovu J, Nyamukapa C (2002). Methods to reduce social desirability bias in sex surveys in low-development settings: Experience from Zimbabwe. Sex Transm Dis.

[pmed-0040102-b019] Gregson S, Nyamukapa C, Garnett GP, Mason PR, Zhuwau T (2002). Sexual mixing patterns and sex-differentials in teenage exposure to HIV infection in rural Zimbabwe. Lancet.

[pmed-0040102-b020] Latif A (1996). Syndromic management of sexually transmitted disease.

[pmed-0040102-b021] Ray CS, Mason PR, Smith H, Rogers L, Tobaiwa O (1997). An evaluation of dipstick-dot immunoassay in the detection of antibodies to HIV-1 and HIV-2 in Zimbabwe. Trop Med Int Health.

[pmed-0040102-b022] Gregson S, Mason PR, Garnett GP, Zhuwau T, Nyamukapa C (2001). A rural epidemic in Zimbabwe? Findings from a population-based survey. Int J STD AIDS.

[pmed-0040102-b023] Gregson S, Garnett GP, Nyamukapa CA, Hallett T, Lewis JJC (2006). HIV decline associated with behaviour change in eastern Zimbabwe. Science.

[pmed-0040102-b024] Gregson S, Mushati P, White PR, Mlilo M, Mundandi C (2004). Informal confidential voting interview methods and temporal changes in reported sexual risk behaviour for HIV transmission in sub-Saharan Africa. Sex Transm Infect.

[pmed-0040102-b025] Bennett S, Parpia T, Hayes RJ, Cousens S (2002). Methods for the analysis of incidence rates in cluster randomised trials. Int J Epidemiol.

[pmed-0040102-b026] Hayes RJ, Bennett S (1999). Simple sample size calculation for cluster-randomized trials. Int J Epidemiol.

[pmed-0040102-b027] Mason PR, Gregson S, Gwanzura L, Cappuccinelli P, Rapelli P (2001). Enzyme immunoassay for urogenital trichomoniasis as a marker of unsafe sexual behaviour. Epidemiol Infect.

[pmed-0040102-b028] Mason PR, Fiori PL, Cappuccinelli P, Rapelli P, Gregson S (2005). Seroepidemiology of Trichomonas vaginalis in rural women in Zimbabwe and patterns of association with HIV infection. Epidemiol Infect.

[pmed-0040102-b029] Mundandi C, Vissers D, Voeten HACM, Habbema JDF, Gregson S (2006). No difference in HIV incidence and sexual behavior between migrants and non-migrants in Manicaland, Zimbabwe. Trop Med Int Health.

[pmed-0040102-b030] Mwaluko G, Urassa M, Isingo R, Zaba B, Boerma JT (2003). Trends in HIV and sexual behaviour in a longitudinal study in a rural population in Tanzania, 1994–2000. AIDS.

[pmed-0040102-b031] Grassly NC, Garnett GP, Schwartlander B, Gregson S, Anderson RM (2001). The epidemiological context and the effectiveness of interventions to prevent HIV in lower income countries. Bull World Health Organ.

[pmed-0040102-b032] Kerrigan D, Ellen JM, Moreno L, Rosario S, Katz J (2003). Environmental-structural factors significantly associated with consistent condom use among female sex workers in the Dominican Republic. AIDS.

[pmed-0040102-b033] UNAIDS (2005). Evidence for HIV decline in Zimbabwe: A comprehensive review of the epidemiological data.

[pmed-0040102-b034] Garnett GP, Anderson RM (1995). Strategies for limiting the spread of HIV in developing countries: Conclusions based on studies of the transmission dynamics of the virus. J Acquir Immune Defic Syndr Hum Retrovirol.

[pmed-0040102-b035] Nyamukapa CA, Hallett T, Mupambireyi Z, Dube S, Campbell C (2005). Interpretation of intervention trial results: Qualitative and mathematical modelling investigations.

[pmed-0040102-b036] World Bank (1997). Confronting AIDS: Public priorities in a global epidemic.

[pmed-0040102-b037] Lamptey P (2002). Reducing heterosexual transmission of HIV in poor countries. BMJ.

[pmed-0040102-b038] Kelly JA, Murphy DA, Sikkema KJ, McAuliffe TL, Roffman RA (1997). Randomised, controlled, community-level HIV-prevention intervention for sexual-risk behaviour among homosexual men in US cities. Lancet.

[pmed-0040102-b039] Woelk G, Kasprzyk D, Montano DE, Mutsindiri R (2002). National survey of STDs and HIV prevalence among residents in rural growth point villages in Zimbabwe.

[pmed-0040102-b040] Voluntary HIV-1 Counselling and Testing Efficacy Study Group (2000). Efficacy of voluntary HIV-1 counselling and testing in individuals and couples in Kenya, Tanzania, and Trinidad: A randomised trial. Lancet.

[pmed-0040102-b041] Cowan FM, Hargrove JW, Langhaung LF, Jaffar S, Mhuriyengwe L (2005). The appropriateness of core group interventions using presumptive periodic treatment among rural Zimbabwean women who exchange sex for gifts or money. J Acquir Immune Defic Syndr.

