# Supplementary material for: Impact and Process Evaluation of Integrated Community and Clinic-Based HIV-1 Control: A Cluster-Randomised Trial in Eastern Zimbabwe
Source: PLoS Med. 2007 Mar 27;4(3):e102. doi: 10.1371/journal.pmed.0040102 (PMC1831737; doi:10.1371/journal.pmed.0040102)
Supplement: Protocol S1 — (35 KB DOC) [file pmed.0040102.sd001.doc]

**Scientific trial of a combined behavioural and STD-control intervention to limit the socio-demographic impact of the HIV-1 epidemic in rural areas of Zimbabwe**

RESEARCH PROTOCOL

***Background and Literature Review***

The Spread and Early Demographic Impact of HIV in Zimbabwe

High levels of HIV prevalence and incidence have been recorded in Zimbabwe since sentinel surveillance was introduced in 1990. Prevalence levels in excess of 40% have been recorded among women attending antenatal clinics in a number of urban centres [1,2]. In a cohort study of male factory workers conducted in Harare between 1993 and 1995, 19% of participants were found to have the infection at enrolment and an incidence rate of 2.93% per annum was recorded [3]. However, rural areas - where 70% of the country's population are based - are experiencing some of the highest rates of incidence at the current time. In Mberengwa, Midland Province, HIV prevalence among pregnant women increased from under 8% in 1992 to 25% in 1994 [4].

In a study in two rural areas of Manicaland Province, we also found high levels of HIV infection at antenatal clinics in 1993-94 - 24% and 14%, at the growth points in the Honde Valley and the Rusitu Valley, respectively. Further data from the Honde Valley in 1996 indicate that incidence remains high (2-4%). Significant increases in adult and infant mortality and in orphanhood were recorded. The cause-specific and age-patterns of increase indicate that these are due primarily to HIV infections. Sexual intercourse with casual partners is common and condom use is low. In qualitative studies, high proportions of men, but relatively few women, reported recent casual partnerships. Mathematical model simulations indicate that this pattern of behaviour could explain the greater excess mortality that was recorded among males, given a recent - last 4-5 years - escalation of the HIV epidemic in rural areas. Other ulcerative and non-ulcerative sexually transmitted diseases (STDs) are common, but only 2% of respondents were aware that STDs can facilitate HIV transmission [5‑8].

Mathematical model projections, based on our understanding of the predominant behaviour pattern and fitted to observed trends in HIV prevalence, also indicate that further increases in adult and early childhood mortality are to be expected. Results from these simulations and empirical findings from Uganda suggest that these increases will result in adverse indirect effects, including high proportions of children experiencing orphanhood and distortions in the age- and sex-distribution of the population [9,10]. These effects may be compounded by fertility change. Birth rates are declining in rural areas of Zimbabwe at present and the HIV epidemic may re-enforce this decline through changes in behaviour or underlying fecundity [11]. Women with HIV infection are reported as having lower fertility than other women in Ugandan studies [12] and results from our research in Manicaland indicate that behaviour changes which would tend to re-enforce the existing decline in birth rates may be beginning to occur [7].

HIV Prevention Strategies

Mathematical model simulations have demonstrated the potential for reducing the incidence of new HIV infections through biomedical and behavioural interventions. The benefits of early interventions and of targeting interventions at core groups have been emphasized [13,14]. Targeted interventions are liable to be less effective if introduced after high levels of HIV prevalence have been reached. Their success will also depend on the level of heterogeneity in sexual activity which exists within a population and the predominant pattern of mixing between different risk groups [15]. Combinations of intervention approaches can act additively and even synergistically [16].

In one set of simulations, based on epidemiological data from rural Uganda, it was demonstrated that a combined biomedical and behavioural intervention which (a) increased condom use in contacts between men and casual sex partners to 50%, (b) reduced the frequency of these contacts by 50%, and (c) reduced the average duration of STD episodes by 50%, could reduce HIV incidence by 50% in the first year and 80% over ten years. Empirical studies have shown that changes of this nature are possible and can lead to large reductions in HIV incidence. In Nairobi, health education and condom promotion led to an increase in condom use among commercial sex workers from 8% to 66%. Condom use was associated with a 3-fold reduction in risk of sero-conversion [17]. In Zimbabwe, large-scale condom promotion programmes were associated with 48% and 53% reductions in STD cases over three years, in Bulawayo and Mutare, respectively. Most of these reductions were achieved within the first year of the intervention [18]. In a study of 752 male factory workers in Mwanza, Tanzania, counselling at an STD clinic contributed to significant reductions in reported high-risk behaviour - eg: the proportion with more than one sexual partner in the preceding month fell from 22% to 12% - and a large (63%) - but non-significant - reduction in HIV incidence [19,20]. In a randomized control trial in rural areas around Mwanza, improved diagnosis and treatment of STDs was associated with a 42% reduction in the incidence of new HIV infections over a two-year period [21,22].

While these examples demonstrate the effectiveness of individual interventions, there remain shortages of scientific evaluations of combined approaches and of programmes which are sustainable within rural communities, where the majority of sub-Saharan African populations are based and where HIV is now spreading rapidly [23]. Studies of this kind are essential to establish the effectiveness of different intervention strategies and to provide demonstration models which can be used to promote the implementation of interventions which are appropriate and cost-effective.

Care and Support Interventions

As HIV epidemics begin to cause higher morbidity, mortality, orphanhood and socio-economic hardship, affected communities require interventions which provide support in coping with these problems, particularly so, as institution-based solutions become less viable. This need is often upper-most in people's minds, so that prevention programmes which are linked to care initiatives - especially where infected community members are supported and involved in prevention - are considered likely to be most effective [24]. Few systematic evaluations have been conducted to assess the effectiveness of existing care and support models. However, it is clear that programmes which include a high level of external biomedical input are unlikely to be financially sustainable [25]. On the other hand, early evaluations indicate that initiatives which encourage local community ownership and participation, whilst more limited in scope, can be extremely cost-effective [26].

Community-Based, HIV Prevention, Care and Support Intervention Strategy for Rural Zimbabwe

In the light of the above, it is proposed to initiate a community-based, combined HIV prevention, care and support intervention in the Honde Valley and neighbouring areas of the Mutasa District of rural Manicaland. The proposed intervention will ultimately cover an area of approximately 2,750 square kilometres, comprising a population of around 170,000 people. The project will be a collaborative programme promoted by Family AIDS Caring Trust (FACT) and the Biomedical Research and Training Institute (BRTI) and involving local community and church groups and local Ministry of Health personnel. FACT is a Zimbabwean NGO based in Mutare, the provincial capital of Manicaland, and has a well-deserved reputation as an organization which has pioneered innovative community-based HIV control activities in Zimbabwe. Its' established projects include prevention and support programmes in both urban and rural areas of the province. FACT will be responsible for behavioural (including condom promotion and distribution activities and community-based promotion of STD services), support and care aspects of the programme. BRTI is a relatively recently-formed organization based in Harare with strong links with the University of Zimbabwe Medical School, the Blair Research Institute and other parts of the Ministry of Health and Child Welfare. BRTI has considerable expertise in STD diagnostic methods and treatment regimes and would be responsible for this aspect of the intervention and evaluation, in close co-operation with the Ministry of Health in Manicaland. It serves a similar function in established HIV control programmes elsewhere in Zimbabwe. BRTI would also act as employer for staff engaged to carry out the fieldwork for the intervention evaluation.

PLAN International have recently extended their area of operation in Zimbabwe to include the Honde Valley and have agreed to provide funding for the HIV intervention programme for a minimum period of four years. PLAN maintain routine records on a number of key impact indicators so that the projects they sponsor can be assessed. It may be possible to develop these systems so that they provide information on indicators of the impact of the HIV epidemic and the effectiveness of AIDS control activities - eg: programme coverage and success in targeting - which could be used in this study and for long-term monitoring purposes.

The proposed intervention strategy appears to have good potential for sustainability and replication. Factors which support this view include the emphasis on local community involvement, the low cost of external inputs, the well-established local NGO partner, Ministry of Health support, and the presence of a rural-based, community-orientated funding organization with a substantial international funding base and a long-term commitment to extending the scope of its activities in Zimbabwe. Descriptions of the principal intervention elements are contained in separate documents attached to this proposal.

***Objectives of the Study***

The principal objectives of the study are as follows:

1. To scientifically evaluate the impact and cost-effectiveness of a combined behavioural and STD-control intervention strategy to slow the spread of HIV infections in rural Zimbabwe; and

2. To extend recent monitoring of the demographic impact of the HIV epidemic in a rural area of Zimbabwe.

Subsidiary aims include validation of innovative methods for data collection on HIV, other STDs - ie: using saliva samples [35] - and sexual behaviour in rural settings (all subject to final pre-test in the pilot study) and for estimating HIV incidence from cross-sectional prevalence data [27]; evaluation of antenatal clinic data as a source of information on HIV levels within the general population; and development of methods for routine evaluation of community-owned support programmes for individuals and families affected by heightened experience of chronic morbidity and mortality.

***Methodology***

Experimental Design for the Intervention Trial

Six pairs of sub-communities (clusters), matched for similar levels of key factors affecting the spread of HIV, will be identified in the vicinity of the Honde Valley. Four of these pairs are likely to be within commercial estates - tea, coffee, forestry and orchards - and two in communal farming areas. Each sub-community will be chosen so that it includes a health clinic where improved STD services can be made available. The behaviour and STD-control intervention will be introduced in one cluster within each pair on a phased basis over a period of twelve months - ie: one new cluster every two months. In each case, a baseline survey for the evaluation will be conducted in the intervention and comparison clusters in the two-month period immediately prior to the introduction of the programme. For the evaluation, 1,000 respondents will be enrolled within a sector of each sub-community following an initial household census - half of those enrolled will be men aged 17-49 years and half will be women aged 15-39 years. Individuals who usually reside in the household will be eligible for full participation in the study – i.e.: recruitment will be on a *de jure* basis - in order to increase follow-up and reduce within-group heterogeneity. Within a household, the random selection process used will avoid instances where a husband and wife or more than one wife are enrolled and thereby reduce correlation of outcomes within individual clusters. Respondents in each pair of clusters will be surveyed again two years months after the baseline visit. Records will be kept on an anonymous basis but a mechanism will be put in place to enable the data to be linked within and between survey rounds.

Outcome indicators will include knowledge about HIV and AIDS, sexual behaviour indicators - partner acquisition and partnership duration, mixing patterns and condom use - incidence of HIV and selected STD infections, and health-seeking behaviour indicators. Sample sizes were calculated to give an 80% power at the 95% significance level, comparing the matched intervention and comparison clusters, for a range of different outcome variables. The formulae used in these calculations take account of possible between-cluster and - in the case of prevalence - within-cluster variability [22,28]. Given the level of spatial mobility which exists within the Honde Valley area, some diffusion effect is likely to be present. This has been reflected in the sample size computations through the use of conservative anticipated excess reductions in the intervention clusters. We estimate that a sample size of 1,000 individuals per cluster will yield a minimum of 700 observations, after accounting for loss to follow-up. In the case of the HIV incidence calculations, these observations will provide 1,120 susceptible person-years of observation, after allowing for a baseline prevalence in the general adult population of 20%. Under these conditions, an initial sample size of 1,000 per cluster should be sufficient to permit detection of a 40% excess reduction in HIV incidence, given a baseline annual incidence of 2% or higher. This sample size should also enable us to detect, *inter alia,* a 40% lower incidence of new HSV2 and trichomoniasis cases among individuals not infected at baseline and a 40% reduction in reported casual relationships without the use of condoms, given a baseline prevalence of 10% or over. Evidence from model simulations and empirical studies suggests that the excess reduction in HIV incidence required is plausible given a combined intervention strategy [19,29]. Furthermore, the required excess reductions in STDs and high-risk behaviour should be attainable. Reductions in these factors, of the order indicated, would, if demonstrated, represent strong indirect evidence that the intervention strategy is slowing the spread of HIV infections. Reduction in other STDs would be a significant benefit, in itself, given the morbidity and adverse fertility consequences they frequently cause.

Data Collection: Quantitative and Qualitative Methods

Quantitative data will be collected on the intervention trial outcome measures summarized above, principally through the two-round survey. A cohort of participants for this survey will be recruited in a household census within each of the twelve study areas. The second round of the study will be conducted two years after the first visit in each area and will involve a repeat household census and further detailed interviews with the original participants. The population-based survey will also provide data on process indicators, including measures of contact with the intervention programme - eg: numbers of meetings attended and visits received from programme personnel - and utilization of programme services - eg: free condoms, revolving loans, care support, STD diagnosis and treatment - and other potential determinants of key outcomes, including socio-economic and demographic factors, and personal experience of HIV-related morbidity and mortality. Data will be collected on demographic indicators - eg: age- and gender-specific mortality, recent fertility, orphanhood and household composition - in the household and individual interviews, using standard demographic methods [30,31]. Quantitative data will also be collected on HIV prevalence at antenatal clinics, so that the representativeness of data from this source can be evaluated. Qualitative data on sexual and health-seeking behaviour and obstacles to behaviour change will be collected in focus-group discussions, pocket-chart voting sessions and key-informant interviews [32,33] and employed to aid questionnaire design and validation and in interpretation of statistical results. These methods were used successfully in our previous study [7]. However, to strengthen this aspect of the current proposal, additional input on the design and implementation of appropriate ethnographic methods will be received from a collaborator from the LSE (Dr. Campbell).

Steps will be taken to develop the good working relationship already formed with the Honde Valley community, to mobilize community support for the research and provide reassurance that information given will be treated as strictly confidential. Thus, we hope to achieve high participation and follow-up rates and more reliable information. Preliminary meetings will be held with community leaders to explain the purpose of the study and the research procedures. Similar information will be given to each prospective participant prior to their decision on enrolment. Data collection methods such as self-completion questionnaires - 96% and 45% of women aged under 50 years, living in the Honde Valley, have received primary and secondary education, respectively, and literacy levels among men are higher than those for women [7] - and secret balloting, which avoid the need for participants to reveal personal information to enumerators directly and thus reduce under-reporting of sensitive information [34], have been field-tested and will be pre-tested further in the pilot study and used where feasible. Similarly, the possibility of using less invasive media such as saliva, urine or blood spot samples for HIV and STD testing is being investigated with the Public Health Laboratory in Harare [35]. The reliability and acceptability of these methods as applied in rural settings will also be field-tested in the pilot study.

Questionnaires will be translated into the local *Manyika* dialect of *Shona*, back-translated and pre-tested in the pilot study. Data entry and validation will be undertaken using SPSS-PC+. Appropriate data entry forms will be designed for each section of the survey questionnaires and skip-rules, range checks etc will be employed to reduce data entry errors. BRTI/Blair staff have considerable experience of using SPSS-PC+ for data entry.

Data Analysis: Scope and Methods

Reliability and validity checks will be conducted to assess the quality of the quantitative data [36]. Data analysis will be conducted using statistical software - eg: STATA and SAS - and mathematical models of HIV incidence, which are based on underlying biomedical, behavioural and demographic determinants of HIV infections [37,38]. Statistical analyses will include tests for the significance of differences arising between paired intervention and control clusters [39,40] and tests for impact of contacts with the intervention programme and other possible determinants on changes in reported behaviour. The impact of the HIV epidemic on mortality will be monitored using longitudinal survey data and official vital registration records. Impact on fertility will be assessed by comparing birth rates among infected and uninfected individuals, controlling for other key determinants including age, education, religion and history of STDs. Crude birth rates, crude death rates and levels of natural population increase will be estimated from the survey data and the trends evaluated [12]. Coverage and impact of care/support interventions will be investigated. HIV prevalence estimated from the population-based survey will be compared with estimates from women attending antenatal clinics to assess the representativeness of the latter. Adjustments will be made in data analysis for the matched-pair design of the study. Data from the study will be used to evaluate new methods for estimating incidence from cross-sectional HIV prevalence surveys [27].

Ethical Considerations

Informed consent will be sought as a condition for enrolment in the study. This will be in written form wherever literacy levels allow - which is expected to be in the great majority of cases. A written statement in the local dialect of *shona* will be provided explaining the purpose of the study and all aspects, benefits and risks of participation. The statement will make it clear that participants have the right to withdraw from the study at any time. Time will be allowed for prospective participants to read the statement, to ask questions and consider their decision. The statement will be read aloud to prospective participants who are unable to read themselves. Enumerators will be trained to do this in a balanced manner so as to avoid possible coercion.

Participants will be given the opportunity to receive HIV-1 test results. Preliminary counselling will be offered during study interviews. Where participants indicate that they would like to be advised as to their own HIV-1 serostatus, appointments will be arranged for them to visit a local clinic where a free counselling and testing service will be made available. Standard Ministry of Health and Child Welfare and WHO procedures will be followed throughout, with further serological tests being undertaken as required.

Participants who test positive for other sexually transmitted diseases will be advised of their results and a free treatment service will be offered wherever possible (eg: for trichomoniasis). Less invasive media such as urine and saliva will be used for the collection of samples for HIV-1 and STD testing. Where blood samples are sought, blood spots rather than venipuncture will be used. Appropriately qualified and trained personnel will be employed to collect the samples.

Rigorous procedures will be maintained throughout the study to ensure full confidentiality of information obtained from participants. Data protection and anonymization procedures will be implemented and computerized matching methods will be used to ensure that access to linked data will be strictly limited to the principal investigators only, with the single exception that appropriate medical staff will be given access to results of HIV-1 or STD tests where participants wish to be informed of their results or to be offered treatment. The procedures will be such that staff, study participants and other individuals are not able to deduce individual HIV-1, STD and sexual behaviour details by applying exclusion and inclusion criteria. Confidential voting methods will be employed in the collection of sexual behaviour information so as to avoid the need for participants to impart personal information to enumerators.

Benefits to participants will include participation in prevention, care and support activities in the intervention areas (see separate description of intervention activities). Subject to confirmation that these activities are effective, the researchers and programme managers are committed to ensuring that similar programmes will be introduced within each control area two years after the study is initiated in that area. In all areas, participants will be offered free treatment of STDs identified in the survey. Free information sheets on HIV-1 infection and AIDS which address gaps in information identified in the earlier study in Honde Valley will be made available to study participants in all areas during the baseline survey. Risks of participation in the research team or as a community member are believed to be low. Rigorous procedures are to be followed to avoid needle-stick injuries and to ensure confidentiality of information obtained, standard treatments will be offered for STDs diagnosed and established tests and associated procedures, which have proved to be very reliable in Zimbabwean settings, will be used for HIV-1 diagnosis, in particular, where results are to be made available to participants.

Dissemination of study results will be done through the Biomedical Research and Training Institute (BRTI) and Blair Research Institute with approval from the Ministry of Health and Child Welfare. An original copy of the data set from the study will remain in Zimbabwe at BRTI and/or Blair. Approval for the study will also be obtained from the Central Oxford Research Ethics Committee in the United Kingdom prior to implementation.

Personnel, Workplan and Location of Project Activities

Dr. Gregson will manage and co-ordinate the proposed research project with expert epidemiological and statistical support from the Blair Research Institute in Harare and the Wellcome Trust Centre for the Epidemiology of Infectious Disease, at Oxford University (Dr. Chandiwana, Professor Anderson, Dr. Garnett and Dr. Donnelly), social psychology and ethnographic support from BRTI, the London School of Economics and the University of Natal (Mr. Tom Zhuwau, Dr. Cathy Campbell, Professor Eleanor Preston-Whyte and Professor Linda Richter), and biomedical support from BRTI (Professor Mason and Mr. Ocean Tobaiwa). Within the team we have considerable management and administrative as well as research experience and in a previous project we demonstrated the ability to develop and conduct a successful fieldwork-based scientific research project, given the level of technical and logistical support envisaged in this proposal. Zimbabwe has a good supply of well-qualified personnel in most disciplines and staff with appropriate sociological, demographic and/or medical expertise will be recruited. All staff will be given in-depth training in the specific requirements of the study. A summary schedule of project activities over time is attached.

***Intended Use of Results***

The results will be disseminated through seminars, workshops and publications in local and international journals. In these ways, we will seek to promote greater awareness of the level of effectiveness of the intervention strategy evaluated in the given context.

Harare

4 July 1997

***References***

1. National AIDS Co‑ordination Programme "*HIV and AIDS Surveillance Annual Report*," Zimbabwe Ministry of Health, 1994.

2. L'Herminez M "*HIV sero surveillance, Masvingo Province, 1995*," Epidemiology and Disease Control, Ministry of Health, Masvingo Province, Zimbabwe, 1996.

3. Mbizvo MT, Machekano R, McFarland W *et al.*: **HIV seroincidence and correlates of seroconversion in a cohort of male factory workers in Harare, Zimbabwe.** *AIDS* 1996, **10**: 895‑901.

4. Van den Hombergh H "*HIV sero‑status survey in Midlands province ‑ phase 1‑3*," Ministry of Health Epidemiology and Disease Control, Midlands Province, 1996.

5. Greenblatt RM, Lukehart SA, Plummer FA *et al.*: **Genital ulceration as a risk factor for human immunodeficiency virus infection.** *AIDS* 1988, **2**: 47‑50.

6. Bassett MT, McFarland WC, Ray S *et al.*: **Risk factors for HIV infection at enrolment in an urban male factory cohort in Harare, Zimbabwe.** *Journal of Acquired Immune Deficiency Syndromes and Human Retrovirology* 1996, **13**: 287‑293.

7. Blair Research Institute, Oxford University "*The Early Socio‑Demographic Impact of the HIV‑1 Epidemic in Rural Zimbabwe*," Blair Research Institute, 1996.

8. Gregson S, Zhuwau T, Anderson RM, Chandiwana SK "*The Early Socio‑Demographic Impact of the HIV‑1 Epidemic in Rural Zimbabwe: Summary Report of Findings from the Manicaland Study of HIV‑1 and Fertility*," Blair Research Institute and Oxford University, 1996.

9. Hunter SS: **Orphans as a window on the AIDS epidemic in sub‑Saharan Africa: initial results and implications of a study in Uganda.** *Social Science and Medicine* 1990, **31**: 681‑690.

10. Barnett T, Blaikie P: *AIDS in Africa*, Belhaven Press: London, 1992.

11. Gregson S: **Will HIV become a major determinant of fertility in sub‑Saharan Africa?** *Journal of Development Studies* 1994, **30**: 650‑679.

12. Sewankambo NK, Wawer MJ, Gray RH *et al.*: **Demographic impact of HIV infection in rural Rakai District, Uganda: results of a population‑based cohort study.** *AIDS* 1994, **8**: 1707‑1713.

13. Rowley J, Anderson RM, Ng TN: **Reducing the spread of HIV infection in sub‑Saharan Africa: some demographic and economic implications.** *AIDS* 1990, **4**: 47‑56.

14. Moses S, Plummer FA, Ngugi EN *et al.*: **Controlling HIV in Africa: effectiveness and cost of an intervention in a high‑frequency STD transmitter core group.** *AIDS* 1991, **5**: 407‑411.

15. Garnett GP, Anderson RM: **Strategies for limiting the spread of HIV in developing countries: conclusions based on studies of the transmission dynamics of the virus.** *Journal of Acquired Immune Deficiency Syndromes and Human Retrovirology* 1995, **9**: 500‑513.

16. Anderson RM, Garnett GP: **Low‑efficacy HIV vaccines: potential for community‑based intervention programmes.** *The Lancet* 1996, **348**: 1010‑1013.

17. Ngugi EN, Simonsen JN, Bosire M *et al.*: **Prevention of transmission of HIV in Africa: effectiveness of condom promotion and health education among prostitutes.** *The Lancet* 1988: 887‑890.

18. Wilson D, Nyathi B, Lamson N *et al.* "*Community Interventions in Zimbabwe: Analysis of a Multi‑Site Replication*," University of Zimbabwe, 1994.

19. Ng'weshemi JZL, Boerma T, Pool R *et al.*: **Changes in male sexual behaviour in response to the AIDS epidemic: evidence from a cohort study in urban Tanzania.** *AIDS* 1996, **10**: 1415‑1420.

20. Pool R, Maswe M, Boerma T, Nnko S: **The price of promiscuity: why urban males in Tanzania are changing their sexual behaviour.** *Health Transition Review* 1996, **6**: 203‑222.

21. Grosskurth H, Mosha F, Todd J *et al.*: **Impact of improved treatment of sexually transmitted diseases on HIV infection in rural Tanzania: randomised controlled trial.** *The Lancet* 1995, **346**: 530‑536.

22. Hayes R, Mosha F, Nicoll A *et al.*: **A community trial of the impact of improved sexually transmitted disease treatment on the HIV epidemic in rural Tanzania: 1.Design.** *AIDS* 1995, **9**: 919‑926.

23. Family Health International "*Workshop on the status and trends of the HIV/AIDS epidemics in Africa*," 1995.

24. Williams G, Blibolo AD, Kerouedan D: *Filling the Gaps: Care and Support for People with HIV/AIDS in Cote D'Ivoire*, ACTIONAID: Oxford, 1995, Vol. **10**.

25. Woelk G, Hansen K, Jackson H *et al.* "*The costs and quality of community home based care for HIV/AIDS patients and their communities in Zimbabwe*," University of Zimbabwe, 1996.

26. Foster G, Makufa C, Drew R *et al.*: **Supporting children in need through a community‑based orphan visiting programme.** *AIDS Care* 1996, **8**: 389‑403.

27. Gregson S, Donnelly CA, Parker CP, Anderson RM: **Demographic approaches to the estimation of incidence of HIV‑1 infection among adults from age‑specific data in stable endemic conditions.** *AIDS* 1996, **10:** 1689-1697.

28. Hseih FY: **Sample size formulae for intervention studies with the cluster as unit of randomization.** *Statistics in Medicine* 1988, **8**: 1195‑1201.

29. Robinson NJ, Mulder DW, Auvert B, Hayes RJ: **Modelling the impact of alternative HIV intervention strategies in rural Uganda.** *AIDS* 1995, **9**: 1263‑1270.

30. Shryock HS, Siegel JS: *The Methods and Materials of Demography (condensed edition)*, Academic Press: London and New York, 1976.

31. Parirenyatwa CN "*Zimbabwe Demographic Health Survey, 1994*," Zimbabwe Central Statistical Office, 1995.

32. Srinivasan L: *Tools for Community Participation ‑ A Manual for Training Trainers in Participatory Techniques.*, PROWWESS/UNDP: Washington D.C., 1990.

33. Smith PG, Morrow RH:*Methods for field trials of interventions against tropical diseases.* Oxford University Press: Oxford, 1991: 326.

34. Catania JA, Chitwood DD, Gibson DR, Coates TJ: **Methodological problems in AIDS behavioural research: influences on measurement error & participation bias in studies of sexual behaviour.** *Psychological Bulletin* 1991, **108**: 339‑362.

35. Chassany O, Bergmann JF, Mazeron MC *et al.*: **Testing of anti‑HIV antibodies in saliva.** *AIDS* 1994, **8**: 713.

36. Dare OO, Cleland JG: **Reliability and validity of survey data on sexual behaviour.** *Health Transition Review* 1995, **4**: 93‑110.

37. Garnett GP, Anderson RM: **Factors controlling the spread of HIV in heterosexual communities in developing countries: patterns of mixing between different age and sexual activity classes.** *Philosophical Transactions of the Royal Society of London* 1993, **Series B**: 137‑159.

38. Gregson S, Garnett GP, Anderson RM:*The Demographic Impact of Infectious Diseases.* Anderson RM ed., Chapman and Hall: London, 1997: Submitted.

39. Donner A, Klar N: **Methods for comparing event rates in intervention studies when the unit of allocation is a cluster.** *American Journal of Epidemiology* 1994, **140**: 279‑289.

40. Gail MH, Mark SD, Carroll RJ *et al.*: **On design considerations and randomization‑based inference for community intervention trials.** *Statistics in Medicine* 1996, **15**: 1069‑1092.

**APPENDIX I**

**Cluster Size Calculations**

**1. Introduction**

A series of power calculations have been carried out to provide a basis for the decision on the size of population per sub-community (cluster) required to yield an acceptable chance of detecting reductions in HIV and STD incidence and changes in sexual behaviour of an order which would justify the costs of the intervention. The purpose of this note is to summarize the principal considerations involved in this decision and to provide some examples of the results obtained.

**2. Issues and Underlying Assumptions**

The principal issues and underlying assumptions taken into account in the cluster-size calculations were as follows:

2.1 For optimal power in the study, the numbers of individual participants in each cluster should be equal. We will therefore aim to recruit equal numbers of participants in each area.

2.2 Larger numbers of matched-pairs of clusters tend to increase power. However, unless the overall study area is extended substantially, the greater the number of clusters, the more difficult it becomes to limit contamination between intervention and comparison groups. Cost and funder constraints restrict the degree to which the study area can be extended. Given these constraints, it was decided to select six matched pairs of clusters. This number will also permit application of non- parametric tests during data analysis [1].

2.3 In the cluster-size calculations for HIV incidence and the incidence of STDs not treated at follow-up, we employed formulae which can be used to reflect the possible impact of between-cluster variability within matched pairs [2]. The examples attached illustrate the potential effects of alternative levels of between-cluster variability. High levels of between-cluster variability limit chances of detecting significant reductions in outcome variables. Thus, it is important to identify pairs of communities that are well matched.

2.4 Rural areas in Zimbabwe fall into two main categories - commercial estates and communal areas. Estates typically employ large numbers of migrant workers, the sex balance of which depends on the nature of the work involved. In forestry estates, men tend to predominate, while in tea and coffee plantations the sex-ratio of workers is more even. The extent of seasonal work also varies between the different types of estate. Communal areas are characterized by subsistence agriculture and women form the majority of the adult population. In small towns and roadside settlements the sex-ratio again tends to be relatively evenly balanced. The stratification of sub-communities takes account of these socio-economic and demographic variations and other factors which could influence sexual behaviour and STD and HIV transmission. In this way, we will seek to minimize between-cluster variation in the outcome variables.

2.5 Diffusion of the effects of the intervention - ie: contamination - will be more difficult to control in a programme which contains a substantial behavioural component than in programmes which focus on improving access to more effective STD treatment (Appendix II). In addition, there is an ethical imperative to offer treatment wherever possible for STDs identified during the baseline survey - in comparison as well as intervention areas. For these reasons, differences in outcome variables between the intervention and comparison populations will tend to understate the true effect of the programme. Thus, results which indicate a significant effect of the programme will tend to be conservative. However, in order to ensure that there is a good chance of detecting genuine changes brought about by the programme, at the 95% significance level, a high level of power (80%) has been used in the sample size calculations. For similar reasons, we have calculated cluster sizes required to detect differences which are smaller than those which might otherwise be expected to be achieved - given findings from mathematical model simulations and single-intervention studies [3‑5].

2.6 In comparing reductions in prevalence - eg: in behaviour variables and self-reported STDs - it is necessary to make allowance for within-group as well as between-group variability in outcomes [6]. We have therefore employed formulae which can be used to show sensitivity of cluster-size calculation results to differences in within-cluster variability [7]. The potential effects of such variability are illustrated in the examples given. Steps are also being taken in the detailed design of the study to achieve the highest possible level of homogeneity within the study populations - eg: by excluding visitors, short-term residents and low-risk age-groups [8].

2.7 HIV and STD infection rates between spouses and, in the case of polygamous unions, co-wives are likely to be highly correlated. A random process will therefore be used for selecting participants within a household, which will avoid the inclusion of both spouses or more than one wife. Sons and daughters will be eligible, subject to the normal age and residence criteria.

2.8 Other things being equal, a longer follow-up period provides a greater number of person-years of observation and reduces the cluster size required to achieve a given power. However, ethical considerations demand that the intervention programme, if effective, be introduced within the comparison populations at the earliest possible time-point. Furthermore, loss to follow-up is liable to increase over time. A two-year time-period between baseline and follow-up surveys therefore is proposed - a period which is in line with previous HIV-control intervention trials [4] and should be long enough to dilute the effects of treatment of STDs identified in the baseline survey in the comparison populations.

2.9 Loss to follow-up over the two-year fieldwork period has been taken to be of the order of 30% - a level similar to that achieved in a previous intervention trial in rural locations [4]. Aspects of the study design which should increase follow-up rates will include obtaining initial informed consent for follow-up, repeat visits to participants who are temporarily absent, flexible interview times, and limiting the length of questionnaires. Socio-demographic and behavioural characteristics of individuals seen at follow-up will be compared with those for absentees and refusals to evaluate possible bias.

2.10 The formulae used for the cluster-size calculations reviewed here assume that programme outcomes can be assessed by reference to indicators with dichotomous endpoints. While the distributions of outcomes such as numbers of sexual partners, births and so on will be investigated during data analysis, the principal outcome indicators for assessing the impact of the intervention will be variables with dichotomous endpoints.

2.11 Following review of the power calculations, it was concluded that approximately 1,000 individuals would need to be recruited in each cluster in order to provide a reasonable chance of detecting significantly lower HIV incidence in the intervention clusters and significant reductions in other STDs and high-risk behaviour. In each of the figures attached, a line has been drawn to show the extent of heterogeneity between and - where appropriate - within clusters which could be present without jeopardizing chances of detecting the changes indicated, given a cluster size of 1,000.

**3. HIV Incidence**

3.1 Estimates of numbers of individuals required - at recruitment - to enable a lower level of HIV incidence to be detected in the intervention clusters than in comparison clusters are shown in Figure 1. Seperate estimates are illustrated for true differences of 40% and 50%.

3.2 These calculations are based on the assumption that HIV prevalence at baseline among study participants is 20%. HIV prevalence among women at antenatal clinics at Hauna - the "growth point" /rural development centre in Honde Valley - and on one of the neighbouring tea estates in 1993-94 was 24.3% (50/206) and 15.8% (9/57), respectively. In a repeat survey at Hauna in 1996, HIV prevalence at antenatal clinics was found to have increased (31.5% - 45/143). Comparisons of the socio-demographic and behavioural characteristics of women attending antenatal clinics and women of childbearing age in the general population indicate that prevalence within the latter group is somewhat lower [9], but reduced fertility among women with HIV could mean that the differential is small. Even in Zimbabwe, HIV prevalence tends to be lower in less urban areas, so that levels in areas away from the growth point are likely to be lower than those at Hauna. There is currently very little data on recent prevalence levels among men in the general population in Zimbabwe. A baseline prevalence below the 20% figure chosen would result in a gain in the power of the study, a higher figure would give a reduction.

3.3 On the basis of local and national data, we estimate that HIV incidence in the study areas at baseline will lie within the range 2%-3% [10‑12]. Given an intermediate figure of 2.5%, a co-efficient of variation between clusters in the matched-pairs of 0.15 would correspond to true incidence rates varying from approximately 1.75% to 3.25% in the comparison clusters. Where HIV incidence is reduced by 40% in the intervention clusters, this level of variation would correspond to a range of true incidence values of around 1.05% to 1.95%. In each case, these represent greater variations than are likely to be seen in practice.

3.4 The results indicate that a cluster size of 1,000 would provide a good chance for detecting a 40% lower incidence of HIV infections in the intervention areas and an excellent chance for detecting a 50% improvement.

**4. STD Incidence**

4.1 In order to contain the costs of the study it is proposed to limit monitoring of other STIs to testing for HSV-2 and trichomoniasis. These infections were selected on diagnostic and epidemiological grounds (Appendix II and s4.2) and because (subject to confirmation in pre-test) it is believed that reliable results can be obtained using blood-spot samples which are relatively uninvasive and can be obtained in household-based surveys. Samples for haemaglobin analysis by haemacue can be obtained at the same time, so that iron supplements can be offered to individuals with low haemaglobin, providing an added benefit to participating in the study and possibly increasing enrolment levels.

4.2 The choice of HSV-2 and trichomoniasis provides one example of an STI which causes genital ulcers and one of an STI which can result in urethral and vaginal discharge - each of which it is understood can facilitate HIV transmission [13]. Both STDs are highly infectious. No effective and affordable treatment for HSV-2 is available in Zimbabwe, so it will not be possible to offer full treatment following diagnosis in the survey or within the intervention. Thus, differences in infection rates in HSV-2 largely will be influenced by differences in sexual behaviour change, while changes in health-seeking behaviour and improvements in diagnosis and treatment will play a part in slowing trichomoniasis transmission. In both cases, the high level of interaction of the study populations with short-term and returning labour migrants would be expected to result in high infection rates in the absence of control.

4.3 Data on prevalence of STDs in Zimbabwe are scarce, although numbers of symptomatic case reports are high [14]. In a recent study in Harare, trichomoniasis was found to be the most common infection recorded (besides HIV) among women attending antenatal and STD clinics (Appendix II - Table 4). Prevalence in rural areas is believed to be higher than in Harare (probably around 20%). Published estimates of recent HSV-2 prevalence in Zimbabwe are not available, but the level is understood to be of the order of 30% among sexually-active adults (Professor Peter Mason, University of Zimbabwe Medical School - personal communication). Other things being equal, prevalence in younger adults would be expected to be somewhat lower due to the chronic nature of the infection.

4.4 For HSV-2, incidence will be measured among individuals without infection at baseline. The cluster size calculation is similar to that for HIV, so that the estimates given in Figure 1 provide a guide to the baseline incidence levels needed to permit detection of 40% and 50% reductions. If incidence levels were constant by age, 30% prevalence by age 35 - ie: mid-way between ages 15 and 55 - would be equivalent to an annual incidence rate of around 2%. In practice, selective exclusion of high-risk groups due to prior infection will tend to bias observed incidence levels downwards at older ages. However, this effect will be compensated for in the study design by the concentration on younger age-groups where sexual activity rates are higher, high-risk groups are better represented and thus annual incidence rates well in excess of 2% are to be expected.

4.5 Cases of trichomoniasis identified during the baseline survey will be treated. In the cluster-size calculations (Figure 2) it has been assumed that prevalence following the survey will be close to zero. At follow-up, it will be possible to compare levels of incident cases which have arisen since baseline but have not yet been treated, in the intervention and comparison areas. For the purposes of the power calculations the formula used in the HIV incidence-based estimates has been employed [2].

4.6 Given the uncertainty regarding likely levels of re-introduction of trichomoniasis - which will depend upon the extent of mixing with individuals from outside the study areas and the level of acquired immunity to re-infection - and the likelihood that many individuals will seek treatment once disease becomes symptomatic even in the comparison clusters, a range of relatively low rates of (untreated) STD infection have been assumed in the cluster-size calculations (Figure 2).

4.7 It will also be possible to conduct statistical tests for differences in incidence of trichomoniasis just among individuals who were not infected at baseline. Such an approach offers the advantages that it restricts the impact of any false-positive diagnoses at follow-up and limits any dilution of the results due to acquired immunity among those treated. A disadvantage is that it disproportionately excludes members of high-risk groups for STIs including HIV. However, again, the effect of this will be limited by the concentration on younger age-groups.

4.8 On the basis of the calculations undertaken, a cluster size of 1,000 should provide an excellent chance for observing a 40% lower level of STIs in the intervention populations at follow-up, even at low levels of infection in the absence of control (Figures 1 and 2).

**5. Sexual Behaviour Change**

5.1 In our early qualitative research in rural Manicaland, we noted that casual sexual relationships are common, particularly among male labour migrants and non-married women [10]. This pattern has also been observed in other parts of sub-Saharan Africa [15]. In a subsequent pilot study in the area surrounding the Zindi business centre in the Honde Valley, we obtained some quantitative data which confirm this impression and indicate that high-risk behaviour may be more common among men on the commercial estates than among those in communal areas. In total, 17% of men reported a casual relationship within the last four weeks and 12% indicated that they had had a casual partner without using condoms at least once during this period (n=142). These levels are thought to be on the low side as the survey took place during Passover, when some local religious groups abstain from all sexual intercourse, and because the survey took place towards the end of the month, some time after the most recent pay-day for estate workers. Higher levels would also be expected if, as is planned, older individuals are excluded from the study [11], and improved data collection methods are applied.

10% of female respondents reported having been paid for sex within the last four weeks (n=194). 6% said they had had a casual partner without using condoms. The equivalent figures for areas outside the business centre might be expected to be slightly lower, but, again, higher levels can be obtained by focusing eligibility criteria to maximize participation among demographic sub-groups who are more likely to engage in risky activities [8].

5.2 The above estimates have been used as the basis for the cluster-size calculations illustrated in Figures 3 and 4. These indicate that a cluster size of 1,000 should give an excellent chance of detecting even relatively modest reductions in casual partnerships. The between-cluster variability parameter estimates correspond to baseline prevalence estimates ranging from 20% to 30%.

5.3 Early studies give conflicting reports as to the most common modifications in behaviour made in response to HIV. In a study in Tanzania, reduction in casual partnerships was the most frequent action taken and condom use was rare [16]. In contrast, condom use has increased in Kenyan and urban Zimbabwean populations [17,18]. Casual relationships will be less risky if condoms are used on a consistent basis. Therefore, the behavioural indicator of greatest interest is likely to be prevalence of having casual partners without using condoms. Cluster-size calculations using baseline estimates for this indicator - prevalence of 8%-16% - are shown in Figure 4. Larger relative reductions in the frequency of this activity should be attainable in the intervention populations, given that two alternative modifications in behaviour would be reflected. However, even with a 40% excess reduction, a cluster size of 1,000 provides a good chance of detecting the change.

**6. Comparison with the Mwanza Study**

6.1 The proposed statistical design of the study is similar to that of the "Mwanza Study" [2] and the cluster-size calculations yield a very similar result - ie: six matched-pairs of clusters, each with a population size of 1,000 followed for two years. However, there are a number of differences in the conditions for the present study and in the assumptions which have been made. In this section, we review the main differences and use the Mwanza Study design as a yardstick for assessing the overall conservativeness of the design proposed for the current study.

6.2 Both studies are located in rural areas. However, the HIV epidemic has already reached a later stage in the present case and its size appears to be considerably larger than that in rural Tanzania. Recent empirical estimates indicate that current HIV incidence rates in Zimbabwe are higher than those envisaged for rural Tanzania when the Mwanza Study was planned. This situation required us to take account explicitly of the pre-existing level of HIV prevalence in the cluster-size calculations for the proposed study. On the other hand, the higher contemporary level of HIV incidence in rural Zimbabwe means that it should be possible to detect similar reductions with a smaller (net) cluster-size.

6.3 The extent of the diffusion effect of the intervention within comparison populations envisaged in the Mwanza Study was low. This is likely to be a more significant issue in the current context as the overall study area is smaller and it will not be possible to completely restrict access to the behavioural component of the intervention programme. We have sought to address this by selecting more conservative anticipated changes in outcome indicators than were used in the Mwanza Study - ie: 40% reductions compared with 50% - despite the potential additive effects of the combined intervention strategy.

6.5 While it was acknowledged that loss to follow-up would reduce the power of the statistical design in the Mwanza Study, this was not accounted for in the sample-size calculations reported. As this may be quite high over a two-year period - and indeed proved to be so in Mwanza - we have made adjustments in the calculations illustrated here.

6.6 It is difficult to compare the levels of variability which are likely to be present within and between clusters with those seen in the Mwanza Study. Use of a strict *de jure* basis for recruitment of participants should increase homogeneity within clusters. Matching clusters by type of rural community should enable us to restrict between-cluster variability. Other selection criteria are being applied to increase the similarity of clusters within communal areas - eg: inclusion of a business centre with bottle-store, similar distances from towns, growth points and tarred roads etc.

6.7 On balance, we believe that the conservatism of the proposed statistical design is on a par with that used in the Mwanza Study.

**7. Conclusion**

7.1 A randomized control design based on six matched-pairs of clusters, each with 1,000 usually-resident adults in the principal sexually-active age-groups, appears to be optimal for a scientific evaluation of the proposed combined behavioural and STD-control intervention.

Simon Gregson

Christl Donnelly

Geoff Garnett

14 March 1997

**References**

1. Donner A, Klar N: **Methods for comparing event rates in intervention studies when the unit of allocation is a cluster.** *Am J Epid* 1994, **140**: 279‑289.

2. Hayes R, Mosha F, Nicoll A *et al.*: **A community trial of the impact of improved sexually transmitted disease treatment on the HIV epidemic in rural Tanzania: 1.Design.** *AIDS* 1995, **9**: 919‑926.

3. Robinson NJ, Mulder DW, Auvert B, Hayes RJ: **Modelling the impact of alternative HIV intervention strategies in rural Uganda.** *AIDS* 1995, **9**: 1263‑1270.

4. Grosskurth H, Mosha F, Todd J *et al.*: **A community trial of the impact of improved sexually transmitted disease treatment on the HIV epidemic in rural Tanzania: 2.Design.** *AIDS* 1995, **9**: 919‑926.

5. Ng'weshemi JZL, Boerma T, Pool R *et al.*: **Changes in male sexual behaviour in response to the AIDS epidemic: evidence from a cohort study in urban Tanzania.** *AIDS* 1996, **10**: 1415‑1420.

6. Simpson JM, Klar N, Donner A: **Accounting for cluster randomization: a review of primary intervention trials, 1990 through 1993.** *American Journal of Public Health* 1995, **85**: 1378‑1383.

7. Hseih FY: **Sample size formulae for intervention studies with the cluster as unit of randomization.** *Statistics in Medicine* 1988, **8**: 1195‑1201.

8. Gail MH, Mark SD, Carroll RJ *et al.*: **On design considerations and randomization‑based inference for community intervention trials.** *Statistics in Medicine* 1996, **15**: 1069‑1092.

9. Gregson S, Zhuwau T, Anderson RM *et al.*: **Age and religion selection biases in HIV‑1 prevalence data from antenatal clinics in Manicaland, Zimbabwe.** *Central African Journal of Medicine* 1995, **41**: 339‑345.

10. Blair Research Institute, Oxford University "*The Early Socio‑Demographic Impact of HIV‑1 in Rural Zimbabwe*," Blair Research Institute, 1996.

11. Mbizvo MT, Machekano R, McFarland W *et al.*: **HIV seroincidence and correlates of seroconversion in a cohort of male factory workers in Harare, Zimbabwe.** *AIDS* 1996, **10**: 895‑901.

12. Mbizvo MT, Mashu A, Chipato T *et al.*: **Trends in HIV‑1 and HIV‑2 prevalence and risk factors in pregnant women in Harare, Zimbabwe.** *Central African Journal of Medicine* 1996, **42**: 14‑21.

13. Laga M:*HIV infection prevention: the need for complementary STD control.* Germain, Holmes, Piot & Wasserheit (eds.), Plenum Press: New York and London, 1992: 131‑144.

14. National AIDS Co‑ordination Programme "*HIV and AIDS Surveillance Annual Report*," Zimbabwe Ministry of Health, 1994.

15. Lagarde E, Pison G, Enel C: **Knowledge, attitudes and perception of AIDS in rural Senegal: relationships to sexual behaviour and behaviour change.** *AIDS* 1996, **10**: 327‑334.

16. Pool R, Maswe M, Boerma T, Nnko S: **The price of promiscuity: why urban males in Tanzania are changing their sexual behaviour.** *Health Transition Review* 1996, **6**: 203‑222.

17. Ngugi EN, Simonsen JN, Bosire M *et al.*: **Prevention of transmission of HIV in Africa: effectiveness of condom promotion and health education among prostitutes.** *The Lancet* 1988: 887‑890.

18. Wilson D, Nyathi B, Lamson N *et al.* "*Community Interventions in Zimbabwe: Analysis of a Multi‑Site Replication*," University of Zimbabwe, Harare, 1994.


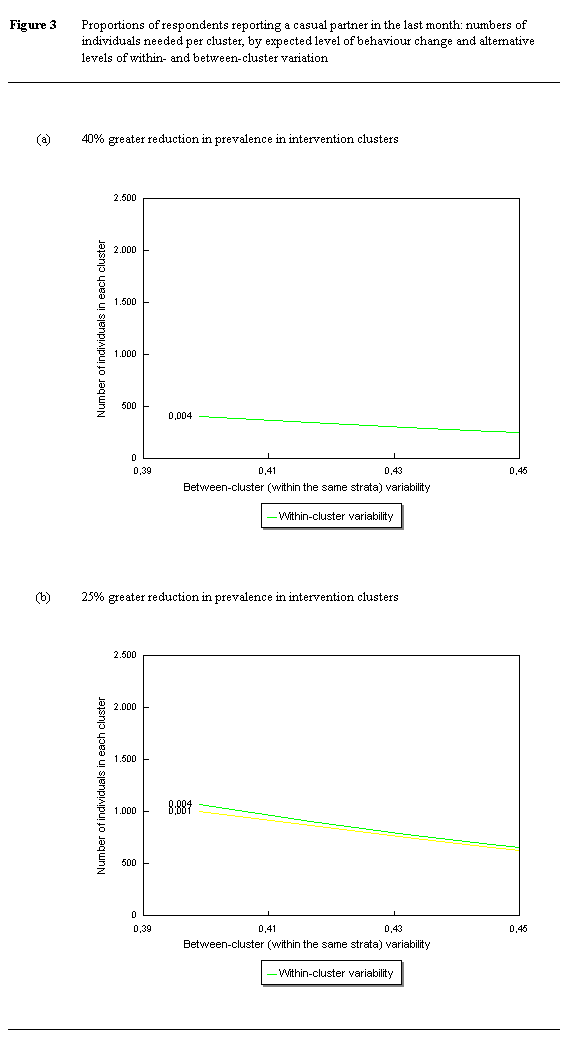


**APPENDIX II**

**Intervention Design and STD/HIV Diagnosis**

**1. Introduction**

This note describes two important difficulties which can hamper attempts to identify genuine intervention effects when utilizing a randomized controlled design in which sub-communities are the unit of analysis in an HIV prevention strategy trial and summarizes the measures to be put in place to limit the impact of these factors in the current study. The first issue is the possibility that intervention effects will also be felt in control clusters so that the effect in intervention clusters is obscured in data analysis ("contamination") [1]. The second is the question as to whether the accuracy of the diagnostic tests used will be good enough to enable genuine differences in the prevalence/incidence of STDs - selected as markers for intervention impact - to be detected, given expected underlying levels of infection and the expected degree of impact of the intervention.

**2. Contamination between Intervention and Control Clusters**

*2.1 Description of the Problem*

2.11 The proposed intervention strategy comprises community-based peer-education activities and strengthening of local STD diagnostic and treatment services. Contamination could occur through participation in peer-education activities or use of STD services in intervention areas by individuals from control clusters. The full benefits of the enhanced STD services might not be felt if individuals from intervention areas seek treatment in other areas due to concerns about privacy.

2.12 The road network in Zimbabwe is good by developing country standards, bus services are reasonable and circular migration is extensive. *Prima facie* the potential for a significant contamination effect is therefore relatively high.

2.13 The above said, the scale of these difficulties should be kept in proportion. In the Mwanza Study in Tanzania, only 0.5 per cent of patients receiving treatment for STDs in the intervention areas were from control clusters [2]. The equivalent percentage would be expected to be higher in the current context but is still likely to be modest. At the same time, local promotion of the strengthened STD services in intervention areas through community peer-education and outreach activities and a strong emphasis on confidentiality and access to same-sex nurses should limit the extent to which services are sought elsewhere.

2.14 Equally, the predominant *pattern* of circular migration from rural areas in Manicaland entails movements to large towns for work (men), petty trading or shopping (women) purposes. Movements to other rural areas are less common, to dispersed locations and tend to be of short duration (Table 1). Exposure to peer-education activities in intervention areas among individuals from control clusters is likely to be limited and for short periods. The focus of these programmes will be on the local community and activities will emphasize regular participation, which has been shown to be associated with safer behaviour [3].

*2.2 Measures Taken to Address the Problem*

2.21 Study sites have been selected so that they extend over a wide area with each cluster lying at least 20 kilometres by road from its nearest neighbour (Figure 1). Most clusters are more than 50km apart and several are separated by mountainous terrain. The shorter distances generally entail travel along dust roads (Table 2).

2.22 Study sites have been selected with a view to minimizing the degree of interaction between cluster populations, with advice and assistance from locally-based Zimbabwe Central Statistics Office officials (Table 3). For example, it is known that there is very little movement between commercial estates or between these estates and communal areas beyond those in the immediate vicinity. People from communal areas rarely travel to growth points and business centres other than to those closest-by and *vice versa*. The sites chosen include two pairs of commercial estates and matched pairs of small towns/growth points/business centres on main roads and outlying communal areas, which are all spatially and socially separate.

2.23 The possibility of contamination was taken into account in the decision to allow for a 20 per cent reduction in HIV-1 incidence in control areas in the cluster size calculations (Appendix I).

2.24 Data on contacts with intervention activities and services within control as well as intervention populations will be collected during the follow-up survey. The home areas of STD clinic attendees will be recorded on a routine basis. These data will facilitate assessment of the relative coverage and impact of programme activities in intervention and control areas and analysis of differentials in impact between matched pairs.

*2.3 Other Factors*

2.31 Extraneous factors such as HIV and STD control activities in other neighbouring areas or the effects of national campaigns or increasing sickness and mortality may also limit the impact of the intervention programme. At present, intensive HIV/STD control activities are only in place in Mutare and Rusape. The possible effects of such factors have been taken into account in the study design through careful stratification of the paired clusters (Table 3), in order to ensure an even impact between intervention and control clusters, and through the assumption of a 20 per cent reduction in HIV-1 incidence in the control clusters. Data will be collected which will permit assessment of the extent of migration into neighbouring areas where peer-education activities are also under way and the extent of use of enhanced STD services available in other areas among individuals in intervention and control areas.

**3. HIV/STI Diagnostics**

*3.1 Description of the Problem*

3.11 The HIV-1 epidemic in Zimbabwe became established in the mid-1980s. Levels of HIV-1 incidence are now probably past the peak. However, the epidemic is a severe one and is more recent in rural areas. As a consequence, incidence levels remain relatively high in rural areas (2 per cent plus). On the other hand, the high level of prevalence anticipated at baseline will reduce the power of the study to detect differentials in HIV-1 incidence. These factors have been taken into account in the cluster size calculations (Appendix I). However, the calculations assume reliable diagnosis of HIV-1 and other sexually transmitted infections. Relatively small errors in diagnosis can jeopardize the chances of detecting significant reductions in infections within intervention areas, particularly where the infections occur at a relatively low rate.

3.12 Highly reliable serological diagnostic tests exist for HIV-1 infection which have been evaluated under Zimbabwean conditions [4]. However, there is a minority population in Manicaland which is unlikely to provide blood samples, particularly where needles similar to those used for vaccinations are employed. Under these circumstances, use of saliva or urine samples could increase participation rates. Diagnostic procedures utilizing these media appear to be reliable in Zimbabwean conditions [5,6] but are more expensive and less well established than serological tests.

3.13 Other sexually transmitted infections are common in Zimbabwe [7] and could be used to validate changes in sexual behaviour [8] as well as to measure overall intervention impact. However, there is an ethical imperative to treat infections identified at baseline wherever this is feasible. Likely levels of re-infection over a two-year period of follow-up are uncertain and could be relatively low. Diagnosis of some of the most common infections (eg: syphilis, Chlamydia and gonorrhoea) is problematic, particularly among individuals with a past history of infection. Test procedures for these infections tend to be either unreliable or expensive.

3.14 New data from a study in Harare (Table 4) suggest that contemporary prevalence levels of active syphilis and of Chlamydia - the infections originally thought likely to be most suitable for use in the study - could be less common than indicated by earlier findings in Zimbabwe and results from neighbouring countries (Appendix I). While incidence of active syphilis and Chlamydia may remain relatively high (eg: in relation to prevalence) due to their acute nature and thus short symptomatic phases, the data suggest that HSV-2 and trichomoniasis could be more viable indicators for a study in Zimbabwe. Both are chronic infections but occur at high levels. Reliable serological tests can be accessed and applied at reasonable expense. HSV-2 cannot be treated in Zimbabwean conditions, so a pool of infecteds would remain within each study population and the level of new infections would be a good marker from which to assess the relative levels of behaviour change in intervention and control populations [9], particularly among those starting sexual activity. An effective treatment for trichomoniasis is available and comparison of levels of this infection would provide a basis for assessing impact of the combined intervention. Trichomoniasis was used successfully as an outcome indicator in a recent evaluation of female condoms in Zimbabwe [10]. Sensitivity and specificity of the IFA test used in the study using culture as the gold standard were 92% and 97%, respectively.

*3.2 Measures Taken to Address the Problem*

3.21 There will be a focus on young adults within the study, among whom HIV/STI incidence rates are currently likely to be highest. This focus will be achieved through the study of rural populations and by employing a lower age limit for participation of 15 years and upper limits of 39 years for women and 49 years for men.

3.22 Study sites have been selected with a view to limiting the numbers of eligible individuals from religions which refuse to utilize medical services and thus to minimizing non-participation if blood samples (blood spots) are to be collected. This is possible as members of these religions tend to be concentrated in particular areas (eg: near Hauna and at Zindi in the Honde Valley).

3.23 Serological tests will be used for HIV-1 and syphilis, HSV-2 and trichomoniasis (each, if included) unless saliva or urine tests are demonstrated to be reliable in the field setting and to give a substantial increase in participation in the pilot study (3.27). If syphilis is included, titres will be examined in the identification of active cases, as standard serological test procedures (RPR/TPHA) may be unreliable.

3.24 Specimens which test negative for HIV-1 at baseline will be confirmed using an alternative method (Abbott ELISA) to minimize the number of false incident cases recorded. All results at follow-up will be confirmed using this assay.

3.25 Where appropriate, analyses will include assessment of STI incidence among individuals confirmed as being not previously infected at baseline, so as to control for the effect of false positives at follow-up resulting from antibodies from earlier (treated) infections. This will also provide a control for possible acquired immunity among individuals infected in the past.

3.26 A field laboratory will be established at a central location with good access to Harare and the study sites, after consultation and site visits. The laboratory will be equipped with refrigerators, a small deep freezer, a centrifuge and a microscope. Two technologists will be employed for specimen handling, examination and storage and one general hand for cleaning glassware etc. The Biomedical Research and Training Institute will take responsibility for preparing a manual of Standard Operating Procedures to be field tested during the pilot phase (3.27). BRTI will also train and employ the field and main laboratory staff, provide laboratory equipment and supplies, carry out Quality Assurance and Quality Control of laboratory investigations, and maintain standard laboratory records.

3.27 A comprehensive pilot study will be undertaken to: (1) field-test sample collection, processing and testing procedures in a rural Manicaland setting; (2) assess participation levels with alternative collection media (ie: venus blood, whole blood using blood spot carriers, saliva and urine); (3) evaluate the reliability of alternative media and testing procedures; and (4) assess levels of incidence of different STIs (including HIV-1, syphilis, Chlamydia, gonorrhoea, HSV-2 and trichomoniasis) within rural Manicaland populations. A final decision as to which STIs to include, which forms of specimens to collect and which testing procedures to be applied will be taken after thorough evaluation of the pilot test results. The evaluation will consider the differentials in levels of incident cases between intervention and control clusters, which are likely to occur and to be detectable for each STI during the study period with the proposed cluster numbers and size (Appendix I). For the time being, it is assumed that the biomedical outcome indicators will be HIV-1, HSV-2 and trichomoniasis and that serological tests will be employed. Urine samples will be collected and stored, so that more reliable LCR tests can be undertaken for Chlamydia and/or gonorrhoea if this subsequently proves to be desirable and affordable.

3.28 HIV-1: blood will be centrifuged at the field laboratory and the sera transferred to four cryovials. Care will be taken to ensure correct labelling. Three vials will be frozen and the fourth kept cool. Field laboratory staff will run ICL Dipstick HIV-1&2 EIAs (over 99.6% sensitivity and specificity [4]) on all sera and will keep combs for a permanent record of reactivity. Training in this will be given in Harare. Each Monday morning, all the sera and the results will be packed and transported by road to Harare. In the main laboratory, all baseline sera negative by Dipstick will be retested with Abbott EIA (100% reliability in tests in the United Kingdom [11,12]). At follow-up, all sera that were negative at baseline will be confirmed by Abbott, whether positive or negative by Dipstick. This will ensure the detection of all seroconverters.

3.29 HSV-2 and trichomoniasis: blood samples will be screened for HSV-2 antibodies by Chiron immunoblots in Harare. This test has proved to be reliable in recent studies at the University Medical School laboratory in Harare. Established serological tests will be used to screen for trichomoniasis [10]. Study participants and their sexual partners will be offered treatment with a single dose 5-nitroimidazole for trichomoniasis in accordance with local EDLIZ guidelines [13].

**4. Conclusion**

The impact of problems of contamination of intervention effects within control clusters and of mis-diagnosis of STIs due to unreliable test procedures in areas of low incidence can be restricted to acceptable levels in the proposed field setting in Manicaland. *Inter alia* this can be achieved through appropriate selection of study sites and STIs. By way of a safety net, a degree of contamination has been provided for in the cluster size calculations (Appendix I). The final selection of STI markers for intervention outcome will be made following expert clinical and epidemiological evaluation of results of a comprehensive pilot study which will establish levels of infection in the study environment and optimal specimen collection and diagnostic strategies within this context.

Simon Gregson

Tom Zhuwau

Peter Mason

14 March 1997

**5. References**

1. Hayes R, Mosha F, Nicoll A *et al.*: **A community trial of the impact of improved sexually transmitted disease treatment on the HIV epidemic in rural Tanzania: 1.Design.** *AIDS* 1995, **9**: 919‑926.

2. Grosskurth H, Mosha F, Todd J *et al.*: **Impact of improved treatment of sexually transmitted diseases on HIV infection in rural Tanzania: randomised controlled trial.** *The Lancet* 1995, **346**: 530‑536.

3. Project Support Group, Psychology Department, University of Zimbabwe "*Community peer‑education to prevent STD/HIV/AIDS among women in Zimbabwe and Zambia*," University of Zimbabwe, 1995.

4. Ray CS, Mason PR, Smith H, Rogers L, Tobaiwa O and Katzenstein DA: **An evaluation of dipstick‑dot immunoassay in the detection of antibodies to HIV‑1 and HIV‑2 in Zimbabwe.** *Tropical Medicine and International Health* 1997, **2**: 83‑88.

5. Chassany O, Bergmann JF, Mazeron MC *et al.*: **Testing of anti‑HIV antibodies in saliva.** *AIDS* 1994, **8**: 713.

6. Ternouth I, Tobaiwa O, Vad K, Barnes D "*A comparison study of saliva versus serum in Zimbabwe*," Zimbabwe Ministry of Health, 1995.

7. Blair Research Institute, Oxford University "*The Early Socio‑Demographic Impact of the HIV‑1 Epidemic in Rural Zimbabwe*," Blair Research Institute, 1996.

8. Quinn TC: **Association of sexually transmitted diseases and infection with HIV: biological cofactors and markers of behavioural interventions.** *International Journal of STD and AIDS* 1996, **7**: 17‑24.

9. Cowan FM, Johnson AM, Ashley R *et al.*: **Antibody to HSV‑2 as serological marker of sexual lifestyle in populations.** *British Medical Journal* 1994, **309**: 1325‑1329.

10. Soper DE, Shoupe D, Shangold GA *et al.*: **Prevention of vaginal trichomoniasis by compliant use of the female condom.** *Sexually Transmitted Diseases* 1993, **20**: 137‑140.

11. Van Kerckhoven I, Vercauteren G, Piot P, van der Groen G: **Comparative evaluation of 36 commercial assays for detecting antibodies to HIV.** *Bulletin of the World Health Organization* 1991, **69**: 753‑760.

12. McAlpine L, Parry JV, Mortimer PP "*An evaluation of sixteen combined anti‑HIV 1/2 screening assays*," Medical Services Directorate, 1993.

13. Zimbabwe Ministry of Health and Child Welfare: *Essential Drugs List for Zimbabwe.* Third ed., Ministry of Health and Child Welfare: Harare, 1994.
