# Supplementary material for: Impact and Process Evaluation of Integrated Community and Clinic-Based HIV-1 Control: A Cluster-Randomised Trial in Eastern Zimbabwe
Source: PLoS Med. 2007 Mar 27;4(3):e102. doi: 10.1371/journal.pmed.0040102 (PMC1831737; doi:10.1371/journal.pmed.0040102)
Supplement: Protocol S2 — (35 KB DOC) [file pmed.0040102.sd002.doc]

**Scientific trial of a combined behavioural and STD-control intervention to limit the socio-demographic impact of the HIV-1 epidemic in rural areas of Zimbabwe**

RESEARCH PROTOCOL

***Subsequent adjustments and refinements made to the original research protocol***

1. The qualifying ages for enrolment at baseline were revised from 17-49 years to 17- 54 years for men and 15-39 years to 15-44 years for women to more broadly represent the primary sexually active age ranges;

2. The sample size per community was reduced from 1,000 to 800 and the period of follow-up for each community was increased from 2 years to 3 years due to funding constraints and slower than anticipated implementation of intervention activities. These changes had no net effect on the power of the study to detect changes in the primary outcome of the study (i.e. HIV-1 incidence);

3. The original protocol did not delineate the outcomes of the study explicitly into primary and secondary outcomes. However, it was always the intention that HIV- 1 incidence would be taken to be the primary outcome as in indicated by its use to determine the sample sizes for the study. More specific measures were developed for the secondary outcomes on sexual behaviour, healthcare-seeking behaviour, and knowledge about HIV/AIDS ^1,2^ during the development of the survey questionnaire;

4. The tests for HSV-2 (at baseline and follow-up) and *T. vaginalis* (at follow-up) have not, as yet, been completed. This is because these tests now have a lower priority given the reduced need to assess the relative contributions of behaviour change and improved STI treatment to change in the primary outcome and because the dried blood spot test for HSV-2 that we proposed to use in the study is no longer being manufactured. In the analysis for the current paper, we used self-reported history of genital ulcers and discharge in the past year as outcome measures for effects of the intervention programme on the incidence of STIs;

5. As stated in the protocol, the specimen collection and laboratory procedure for measuring HIV-1 incidence was developed following an evaluation of alternative possible methods carried out in the pilot study ^2^. This procedure is described in the paper;

6. The proposal to conduct haemoglobin analysis by haemacue on dried blood spots collected in the study was dropped due to failure to secure funding for this procedure.

***Reference***

1. Gregson S, Zhuwau T, Anderson R, Chandiwana S. Is there evidence for behaviour change in response to AIDS in rural Zimbabwe? *Social Science and Medicine* 1998;**46**(3)**:**321-330.

2. Gregson S, Mason PR, Garnett GP, et al. A rural epidemic in Zimbabwe? Findings from a population-based survey. *International Journal of STD and AIDS* 2001;**12:**189-196.
