# Supplementary material for: Impact and Process Evaluation of Integrated Community and Clinic-Based HIV-1 Control: A Cluster-Randomised Trial in Eastern Zimbabwe
Source: PLoS Med. 2007 Mar 27;4(3):e102. doi: 10.1371/journal.pmed.0040102 (PMC1831737; doi:10.1371/journal.pmed.0040102)
Supplement: Text S2 — (2.8 MB PDF) [file pmed.0040102.sd004.pdf]

Oxford Radcliffe HOSPITAL

NURSING & ALLIED PROFESSIONS RESEARCH ETHICS COMMITTEE
THE JOHN RADCLIFFE

Manor House
Headley Way, Headington
Oxford OX3 9DZ

Tel: 01865 222692/222547
Fax: 01865 222699

Our Ref. LC/OB/N97.039

24 September 1997

Dr Simon Gregson
Wellcome Trust Centre for the
Epidemiology of Infectious Disease
Zoology Deparment
Oxford University
South Parks Road
Oxford OX1 3PS

Dear Dr Gregson

Re: NAPREC 97.039 - Scientific Trial of a Combined Behavioural and STD-Control Intervention to Limit the Socio-Demographic Impact of the HIV-1 Epidemic in Rural Areas of Zimbabwe

Thank you for submitting your study to NAPREC. It was discussed in detail at our meeting on the 19 September 1997.

I am happy to grant ethical approval to the study and wish you every success with the study. I would be very grateful if you could send me a copy of any publication which may arise from this study.

You should inform NAPREC of any adverse reactions. In addition, if the investigators do not follow the protocol, or have protocol changes, but fail to inform NAPREC, then the Ethics Committee approval will be withdrawn and will no longer be binding.

Yours sincerely,

Lindsey Coombes

Mr Lindsey Coombes
Chairperson
Nursing and Allied Professions Research Ethics Committee

Telephone: 791792/791193/792747
Telefax: (263) - 4 - 792480
E-mail: mrcz@healthnet.zw

Medical Research Council of Zimbabwe
Josiah Tongogara / Mazoe Street
P. O. Box CY 573
Causeway
Harare

Date **8 December, 1997**

Dear Dr. Gregson

Thank you for your proposal on: "Pilot test of an HIV/AIDS counselling and testing intervention for rural areas of Zimbabwe; Scientific trial of a combined behaviour and STD control intervention"

Which was reviewed and the following recommendations/amendments were made:

N/A

Your proposal was approved/rejected. Reasons for rejection:

Approved

Other:

Please be reminded to send in your research results for our Health Research Database

MEDICAL RESEARCH COUNCIL OF ZIMBABWE
1997 -12- 08
P.O. BOX CY 573
CAUSEWAY, ZIMBABWE

Kind regards from the MRCZ Secretariat.

[signature]
FOR CHAIRMAN, MEDICAL RESEARCH COUNCIL OF ZIMBABWE

cc. Ms Mawindi, Research Council of Zimbabwe

INNOVATIVE RESEARCH IN HEALTH DEVELOPMENT
Executive Committee: *Prof. F.W.G. Hill (Chairman), Dr. P.D. Parirenyatwa, (Vice-Chairman)*
Dr. S.K. Chandiwana (Secretary)

To residents of (study areas)

Blair/BRTI Notepaper
Date

Dear Resident

Manicaland Sexually Transmitted Disease Prevention Study

We are asking you if you would kindly help us with a research study which involves examining trends in sexually transmitted diseases and practices which can increase or reduce the chances that these diseases will spread within the community.

We are doing the study to see which types of prevention activities are most successful in slowing the spread of sexually transmitted diseases including HIV/AIDS.

If you agree to participate we would ask you to help us in these ways:

(1) Answer some questions on general topics and on your views and personal experience regarding health matters and sexual relationships;

(2) Provide blood, urine and saliva specimens□u*□ which we can use to carry out tests for the presence of sexually transmitted infections, some of which can occur without noticeable symptoms. The blood samples will be small (120ml in total - less than a teaspoon) and will be collected from your thumb using a small needle.

We would come back to see you again in two years time when we would need to ask you to help us in the same way. If you decide then that you no longer wish to participate, you will be free to withdraw from the study.

We believe that any risks of participating in the study will be extremely small because new equipment (eg: needles) will be used to collect all blood and saliva samples and careful steps are being followed to make sure that any information you provide will be treated in strict confidence. Personal information (eg: results of medical tests) will only be disclosed to medical staff if you specifically ask for this to be done so that you can receive treatment. Please feel free to discuss any of these arrangements with your interviewer.

The advantages of participation include free tests for a number common sexually transmitted infections. If not treated, some of these can result in unpleasant symptoms and may lead to infertility. Some sexually transmitted infections cannot be cured (eg: HIV) but where infections are discovered for which treatment is available, you will be informed and will be able to receive free treatment at a local health clinic. If you think you might like to know whether you have HIV, you will be entitled to attend a free fully-confidential counselling and testing service. Your interviewer can give you details of this service.

You are free to decide not to take part in the study and, if you join, can leave it at any stage. You would remain entitled to use the normal medical health facilities. We suggest that you keep this letter in case you need to show it to anyone concerned with your medical care.

If you have any questions or problems, please contact us at ... (the study address in Manicaland).

Yours sincerely

Simon Gregson
Tom Zhuwau

Research Co-ordinators

□u□nb: which combination of these specimens will be sought will be determined from the results of the pilot study. Copies of the letter will be available in the local dialect of Shona and, where necessary, the letter will be read aloud to respondents.*

Manicaland HIV/STD Prevention Study

Participant Consent Form

1	(a) *For respondents who can read* : have you read the project information sheet?	Yes<br>No	☐<br>☐
	(b) *For respondents who are not able to read* : have you had the project information sheet read aloud to you?	Yes<br>No	☐<br>☐
2	Have you had the opportunity to ask questions and discuss the study?	Yes<br>No	☐<br>☐
3	Have you received satisfactory answers to your questions?	Yes<br>No	☐<br>☐
4	Have you received enough information about the study?	Yes<br>No	☐<br>☐
5	Do you understand what is involved in participating in the study?	Yes<br>No	☐<br>☐
6	Do you agree to participate?	Yes<br>No	☐<br>☐
7	What is the name of the person who explained the project to you?		____________
8	Do you understand that you are free to leave the study:<br>- at any time;<br>- without having to give a reason for leaving;<br>- and without affecting your medical care?	Yes<br>No	☐<br>☐
9	Signed		____________
10	Name (in capital letters)		____________
11	Witness (interviewer) signature		____________
12	Date		____________
